# Investigating the effectiveness of interventions intended to reduce loneliness using psychological strategies and a theory of change: a systematic review of interventional studies and meta-analysis

**DOI:** 10.1186/s40359-025-03639-3

**Published:** 2025-12-12

**Authors:** Mary Birken, Sarah Ikhtabi, Thomas Steare, Sonia Johnson, Giulia Benedetto, Fiona Lin, Hannah Rachel Scott, Jasmine Harju-Seppänen, Tayla McCloud, Roz Shafran, Alexandra Pitman

**Affiliations:** 1 UCL Division of Psychiatry, London, UK; 2 North London NHS Foundation Trust, London, UK; 3 Unit for Lifelong Health and Ageing, UCL, London, UK; 4 UCL Great Ormond Street Institute of Child Health, London, UK

**Keywords:** Loneliness, Psychological, Effectiveness, Cost-effectiveness, Systematic review, RCT, Trial, Theory of change

## Abstract

**Background:**

The adverse effects of chronic loneliness on mental health are well-established. Psychological interventions are more likely to be effective if based on a Theory of Change (ToC) that clarifies intended mechanisms. We aimed to inform development and implementation of psychological interventions for loneliness by synthesising the literature evaluating the effectiveness/cost-effectiveness of psychological strategies with the primary aim of reducing loneliness, specifying their underlying ToC.

**Methods:**

We searched five electronic databases (CINAHL, PubMed, PsycINFO, Scopus, and Web of Science) from 2016 to August 2023 for studies evaluating the effectiveness and/or cost-effectiveness of psychological interventions that targeted loneliness as a primary outcome and were based on a discernible ToC. We conducted quality appraisal using the Cochrane Risk of Bias tool and used the approach of narrative synthesis to report findings.

**Results:**

Twenty two studies met eligibility criteria, including 2,567 participants, and ten theoretical approaches: Cognitive behavioural therapy (CBT) (*n* = 10), mindfulness (*n* = 4), reminiscence therapy (*n* = 3), social identity theory (*n* = 1), behavioural activation (*n* = 1), self-management of wellbeing theory (SWB) (*n* = 1), imagined interaction theory (*n* = 1), Orem’s self-care deficit theory (*n* = 1), logotherapy (*n* = 1) and interpersonal therapy (*n* = 1). All trials were judged to be at some/high risk of bias. None assessed cost-effectiveness. The ToCs for CBT included i) cognitive reappraisal of maladaptive cognitions, ii) promoting social engagement, and iii) developing coping skills to break reinforcing patterns that maintain loneliness. Pooled effect size (*g* = -0.463,95%CI:[-0.765 to -0.162]) shows CBT moderately reduces loneliness. The ToCs presented for other approaches included targeting self-awareness, improving emotional regulation, and enhancing group belonging. We identified medium strength evidence to support interventions based on *CBT*, *social identity theory*, *reminiscence therapy*, *Orem’s self-care deficit theory* and *logotherapy*. We identified very weak evidence to support interventions based on *behavioural activation* or *mindfulness*. We found no evidence to support interventions based on *SWB theory*, *interpersonal psychotherapy*, or *imagined interaction theory*.

**Conclusions:**

We identified a range of theoretical psychological mechanisms of reducing loneliness. Our summary of these psychological approaches and their underlying ToC provides a valuable resource to researchers involved in intervention development. Methodological limitations of included studies guide the design of larger-scale trials.

**Supplementary Information:**

The online version contains supplementary material available at 10.1186/s40359-025-03639-3.

## Introduction

Loneliness is an important public health target because of its association with poor physical and mental health [1]. It is defined as the distressing and unpleasant subjective state of feeling that one’s social relationships are deficient in terms of quality or quantity [2]. Loneliness can arise even among those who are not objectively socially isolated [2]. Loneliness is multidimensional, with i) emotional loneliness stemming from the absence of an intimate relationship or a close emotional attachment, ii) social loneliness stemming from lack of a feeling of belonging to a broader group of contacts or a valued engaging social network, and iii) existential loneliness stemming from the realisation that one is fundamentally alone [3]. Whilst transient loneliness is a common occurrence after a relationship loss or geographic relocation, and potentially an adaptive motivator for forming social connections [4], chronic loneliness is thought to induce changes in social threat perception and physiology that adversely affect physical and mental health [4].

### Prevalence of loneliness and its association with health outcomes

Population surveys show that loneliness is particularly prevalent among young people and older adults [5, 6]. According to the Office of National Statistics, 27% of adults in the UK reported feeling lonely sometimes, and 7% reported feeling lonely always or often [7, 8]. Epidemiological studies describe associations of loneliness with poor physical and mental health [9, 10] as well as with all-cause mortality [11] and suicide mortality [12]. Of all mental disorders, there is most robust evidence describing the longitudinal association of loneliness with depression [13]. Given these health consequences, the high prevalence of loneliness [10], and estimates of its economic cost [14, 15], loneliness is now a pressing public health concern. The US Surgeon General has identified loneliness as a critical public health issue, emphasising the need for collective action to address loneliness and its adverse effects [16].

It is therefore important that we identify which interventions are most effective and cost-effective in reducing loneliness in defined groups, particularly if they are also associated with wider health gains. The focus of this review is psychological interventions using psychological strategies, and we define such psychological interventions as “a method to improve health by means of strategies that induce changes in a patient's cognitions, emotions, and behaviours according to an explicit psychological theory” [17], p. 402). To design and develop effective psychological interventions addressing loneliness, it is essential that they are underpinned by a clear theoretical framework [18] and target defined groups [19], setting out the psychological mechanisms by which the intervention would reduce loneliness.

### Theory of change in intervention development

The theoretical framework on which any psychological intervention is based should be used to build a theory of change (ToC) describing how an intervention is supposed to work [18]. The ToC sets out the key active components of the intervention, the mechanisms activated by them, the intermediate and final outcomes, and the contextual factors that may have an effect, together helping to examine the contribution of each of those components to outcomes [18, 20]. According to the Medical Research Council (MRC) guidelines on the development of complex interventions, interventions specifying the underlying ToC are more likely to be effective and sustainable because they consider the interaction between the intervention and local context and causal pathways through which an intervention might achieve impact [18, 21]. Theory-driven interventions are also easier to evaluate in that the ToC provides a set of indicators to evaluate on all stages of the causal pathway [18]. Additionally, interventions that articulate the mechanisms involved in their ToC are more valuable in that this information can be used to underpin future intervention development [18]. For this reason, the Cochrane Collaboration advise that evidence reviews include a theoretical explanation for how each intervention works, setting out each ToC as means of describing intended mechanisms [22].

### Existing psychological approaches targeting loneliness

Approaches to addressing loneliness can be broadly categorised into social based approaches (e.g. enhancing social support, increasing opportunities for social interaction, improving social skills) and psychological based approaches [23–25]. The most promising evidence for reducing loneliness in the general population relates to psychological interventions that address cognitions [25–27] with the same suggested for people with mental health problems [24]. Psychological factors are increasingly recognised as important contributors to loneliness and there is therefore great interest in developing interventions targeting them [25, 28, 29].

Our previous literature review of studies describing psychological aspects of loneliness, commissioned by the Campaign to End Loneliness, identified the following (overlapping) factors: attributional style; avoidance; cognitive function and impairment; coping style; emotion regulation; group/social identity and self-identity; mental health problems; personality traits or characteristics; purpose in life; resilience; social cognition; self-esteem, self-confidence, and ‘self-efficacy’; social skills; stigma and self-stigma [30]. Such factors influence loneliness in relation to attitudes to self and others, sense of connection to others, and a sense of belonging [30]. They may act as part of the mechanism by which people begin to feel lonely, remain lonely, and/or by which loneliness has its impact on mental health, and/or serve as barriers to accessing community-based services [30]. This suggests that tackling psychological factors may be more important than other more practical approaches because psychological barriers may prevent people from using social opportunities [27].

A range of psychological approaches have been developed to target modifiable psychological factors implicated in loneliness. Some common psychological approaches include cognitive approaches (addressing factors such as attributional style and maladaptive social cognitions), behavioural approaches (addressing factors such as avoidance), and mindfulness-based approaches (addressing factors such as self-acceptance and awareness) [30]. These psychological approaches might include psychological strategies or methods and techniques such as cognitive restructuring, positive reinforcement, behavioural experiments, social skills training, meditation or goal setting [25, 30]. However, there is a knowledge gap regarding the strength of the evidence for each approach, making it unclear which psychological factors to target and psychological strategies to incorporate into interventions targeting psychological factors [31, 32]. Previous systematic reviews suggest that psychological approaches show promise where delivered to people with pre-existing mental health problems [33], people in the general population [25, 31], older adults [34, 35], and young people [27, 36]. However, these reviews also demonstrate that many trials of interventions to address loneliness are based on vague theoretical principles, lacking clarity on how the intervention might alter the participant’s experience of loneliness [36] or not specific to understanding the processes that lead to or maintain loneliness [31]. Even where interventions are found to be effective, it is unclear which specific processes are involved in implementing a successful intervention [34].

To inform further development in this field, there is a clear need to identify which mechanisms and what overall ToC, if any, underpin psychological strategies for addressing loneliness, and to summarise randomised controlled trial (RCT) evidence on the effectiveness of psychological interventions in which an underlying ToC can be discerned. This is particularly important when identifying the ToC for approaches less established than explicit theoretical approaches such as cognitive behavioural therapy (CBT) [24, 25, 31]. Such an overview of approaches and of their ToC is of value to clinicians and researchers seeking to adapt any interventions identified as effective for specific settings or populations. Our aim was to address this gap in the literature by conducting a systematic review of RCTs evaluating the effectiveness and cost-effectiveness of psychologically-focused interventions designed to target loneliness, based on an explicit or implicit ToC. When summarising findings, we set out to identify in each case the intervention’s ToC (where described by authors or discerned by our team), to enhance the theoretical understanding of the processes involved. This and our strict inclusion criteria, restricted to RCTs specifying loneliness as a primary outcome, was intended to identify the postulated causal pathway for each psychological intervention in targeting loneliness to inform future theory-driven intervention development.

## Methods

### Protocol and search strategy

We followed PRISMA guidelines [37] (See Additional File 1 and 2: Appendices 1 and 2) and pre-registered our protocol on Prospero (PROSPERO 2019 CRD42019136373).

### Eligibility criteria

#### Population

We included participants of any age, sampled from any geographical population, but excluded studies sampling participants with intellectual impairments and dementia. This exclusion was on the basis that their levels of cognitive impairment represented a very specific context for their loneliness, and that those findings might not be transferable to other populations [38, 39].

#### Interventions

We wished to identify evaluations of psychological interventions that focused specifically on internal cognitions and feelings with the aim of reducing loneliness, and that were based on a discernible ToC (whether set out explicitly by the authors or described implicitly through setting out the intended psychological changes or pathways). We classed interventions as having a discernible ToC if studies proposed specific psychological mechanisms (e.g. targeting cognitions/feelings) through which the intervention was hypothesised to reduce loneliness. In some studies, the ToC was explicitly detailed within the study’s theoretical framework. In others, we inferred the ToC from (a) the intervention’s stated activities and goals that focused on impacting psychological factors, or (b) the wider discussion throughout papers regarding the psychological processes that might underpin the intervention’s impact on loneliness.

We excluded studies that solely describe the intervention activities or describe/explain a type of therapy generally such as in the introduction without linking it to loneliness.

We included studies whether the intervention was delivered in a group, one-to-one, or remotely (using the internet or telephone). We included interventions of any duration and frequency. We also included multi-component interventions if one or more component(s) addressed psychological factors, even if other components were focused on non-psychological factors.

#### Comparator

Studies were included where the study participants in the comparator group received treatment as usual, a comparator intervention, or a waiting list control.

#### Outcome

We included studies that specified loneliness as the primary outcome, as measured using a validated tool, such as the UCLA Loneliness Scale [40] or the de Jong Gierveld Loneliness Scale [41]. This restriction was to ensure that findings reflected the primary aim of the intervention being to reduce loneliness. Where studies used more than one loneliness measure as a primary outcome, we focussed on findings relating to validated loneliness measures (but also reported findings on unvalidated measures to provide context).

#### Types of study

We included studies that reported formal evidence of effectiveness and/or cost-effectiveness of psychological interventions to address loneliness in the general population and/or clinical populations. Therefore, we included studies reporting RCTs, whether pilot or full trials in this review, and excluded observational studies due to risk of bias and the potential to overestimate treatment effects.

Studies in any language were included in this review.

### Search strategy

We searched five electronic databases of published studies (CINAHL, PubMed, PsycINFO, Scopus, and Web of Science) and the World Health Organisation (WHO) International Clinical Trials Registry Platform, using the following search terms: psych* OR resilien* whether in abstract, title or keywords AND lonel*, whether in abstract, title, or key words, setting no population limit. Appropriate truncation and word combinations were adapted for each database search.

We used a two-stage search of online databases of published studies. The first stage was a search for systematic reviews of studies evaluating the effectiveness of interventions to reduce loneliness published within the previous ten years to screen their included papers for inclusion in this review. In each database, we selected reviews as the type of document searched for.

The second stage was to search for individual trials evaluating the effectiveness or cost-effectiveness of interventions to reduce loneliness that focused on addressing psychological factors, published from 1 st January 2016 (the date of the most recent studies found in the systematic reviews screened in stage one), until the date of conducting the search. The initial searches for both stages were carried out in March 2019 and an updated search for individual trials was conducted in May 2021. The search was updated again in August 2023.

### Data extraction (selection and coding)

Two reviewers (MB and HS) independently screened all study titles and abstracts for potentially eligible systematic reviews identified in the databases in stage one. Two reviewers (TS and HS) screened the included studies of those systematic reviews to identify eligible trials, initially screening titles and abstracts, and then conducting full text screening. In the second stage, we conducted a search for individual trials published from 1 st January 2016 which was then updated in May 2021 and August 2023. Four reviewers (MB, JH-S, SI and FL) screened title/abstracts of primary studies identified from the databases. In the 2016 and the 2021 update, three reviewers (MB, JH-S and TS) screened titles and abstracts of primary studies identified from the systematic reviews/databases. MB then screened 10% of excluded studies selected at random to check consistency of adherence to the inclusion/exclusion criteria at abstract and title screening stage. MB and TS then independently screened full text papers for eligibility. MB, TS, and AP then extracted data to delineate each intervention’s ToC.

In the 2023 update, two reviewers (SI and FL) screened titles and abstracts of studies identified from the databases. GB then screened 10% of excluded studies selected at random to check consistency of adherence to the inclusion/exclusion criteria at title and abstract screening stage. FL and SI then screened full text papers for eligibility. GB also independently screened all full-text papers. GB, FL, and SI extracted data to delineate each intervention’s ToC. Two reviewers (TM and SI) screened titles and abstracts of registered trials identified in the WHO International Clinical Trials Registry from 2009 to 2023.

Data were extracted for each eligible trial included in this review regarding: study design, population sampled, sample size, description of intervention, control group, primary outcome(s), and evidence of effectiveness and/or cost-effectiveness (e.g. p-value). A separate table was used to extract information on psychological factor(s) targeted, intervention processes and actions, intervention format and ToC. For each paper, one reviewer extracted data, and another reviewer checked/reviewed the data extracted.

We used a PRISMA flow diagram to present the flow of studies in the study.

### Quality assessment

We used the Cochrane Risk of Bias tool, ROB 2, [42] to assess the quality of included studies. The tool outlines five areas for assessment of risk of bias and summarises the scores for each to denote if a study is of low risk of bias, whether there are some concerns, or whether the study is of high risk of bias. Two reviewers assessed each included study (MB and TS) and agreed upon scores for each domain of the tool, and a final score for each study. A third reviewer (AP) was consulted where the two reviewers disagreed on any scores. Studies were not excluded based on their risk of bias, but instead the results of each study were reported in the context of their assessed risk of bias. In the updated search, two reviewers (SI and GB) independently assessed risk of bias using the same process described above.

### Data synthesis

We used the approach of narrative synthesis, as outlined by the Cochrane Collaboration [22], to summarise trials’ findings on effectiveness and cost-effectiveness, identify the underlying ToC, and appraise the trial for any sources of bias, thus interpreting findings in this context.

As a post hoc revision to our pre-registered protocol, although the focus of our review was on interventions’ ToC, we decided it would be useful to convey the strength of the evidence for each category of theoretical approach (where feasible based on numbers and homogeneity of measures) using robust systematic approaches.

For any such feasible category, we conducted a random-effects meta-analysis comparing loneliness outcomes at the end of treatment between treatment and control groups. We focused on CBT interventions only as all other intervention domains included a small number of studies, limiting the usefulness of a meta-analysis for these interventions. We also noted the recent systematic review and meta-analysis by Hickin et al. [31] that had assessed the effectiveness of psychological interventions and types of interventions using broader inclusion criteria. Two authors (TS and SI) independently extracted data on the mean and standard deviation of continuous loneliness measures at the end of the intervention for both treatment and control groups, and the number of remaining participants at this time-point for all relevant papers. Any inconsistencies in data extraction were resolved by a senior author (AP). Effect sizes were calculated as the standardized mean difference (Hedge’s g), enabling cross-study comparisons where the outcome measures differed [43]. We emailed lead authors of any studies for which there were missing data (e.g. on the number of participants at the outcome measurement time-point). We excluded from the meta-analysis any studies using more than one loneliness outcome measure without specifying which was the primary outcome, on the basis that there was no clear rationale for selecting any specific outcome over another. To explore the biases introduced by excluding studies with missing data, we conducted a sensitivity meta-analysis by adding the two studies with missing sample sizes at follow-up, on the assumption that there was no attrition from baseline.

For all other categories of theoretical approach for which the number of studies was > 1 we assessed the certainty of the evidence using the Grading of Recommendations, Assessment, Development, and Evaluations (GRADE) scoring system [44] with GRADE criteria adapted for a narrative synthesis approach [45]. Two researchers (AP and MB) independently assessed the certainty of evidence for each category of theoretical approach and resolved any inconsistencies.

## Results

Our searches identified a total of 11,887 papers following removal of duplicates. This included 88 papers identified from five reviews found in the databases, 11,499 papers found from database searches for studies published since 2016, and a further 300 found in trial registries.

**Fig. 1:**
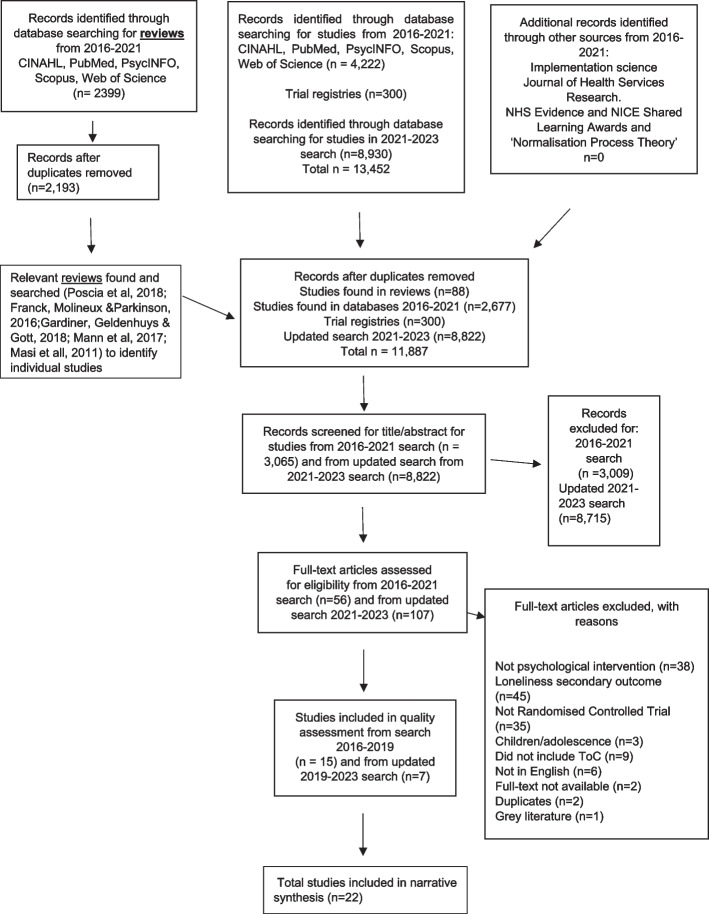
Flow of studies through the review process

Following screening of all titles and abstracts, 163 articles were identified for full text screening. We excluded 141 full text papers (see Fig. 1 for flow of studies through the review process). Initially 11 studies met our eligibility criteria, but following a further search conducted in May 2021 and further search conducted in August 2023, an additional 11 (four and seven, respectively) studies were added, such that 22 studies were included in this review. All the included studies were published in English.

### Study characteristics

Characteristics of the 22 included studies are presented in Supplementary Table 1 (see Supplementary Table 1), which we grouped by type of psychological approach. Included studies were conducted in 11 countries, including eight in the USA and others in China, Israel, Taiwan, South Africa, Sweden, the Netherlands, Australia, Turkey, Iran, and South Asia. One multi-centre study was conducted in India, Burma, Sri Lanka and Nepal [46]. Included studies were published between 1985 and 2023, with 16 studies published in 2019–2023, suggesting growing investment in trials in this area. Trial populations varied in age,with the most common age group being adults over the age of 60 (*n* = 8) [46–53].

The majority of studies (*n* = 11) focused on population samples identifying as having moderate or high levels of loneliness or experiencing distress resulting from loneliness [48, 49, 53–61]. Three of the studies focused on people diagnosed with mental health problems [55, 56, 62], and two focused on people who met criteria for mental health issues [51, 63].

#### Interventions

Twenty-nine interventions were evaluated in the 22 studies; 17 studies compared one intervention to a control, one study evaluated two interventions compared in a four-arm study design [60] whilst four studies evaluated two interventions each in a three-arm study design [55, 58, 62, 63].

The ToC for each intervention is presented in Supplementary Table 2 (see Supplementary Table 2), including the psychological factor(s) targeted and the intervention processes and actions, grouped by theoretical approach. Papers that compared two interventions and provided a description of a ToC that spanned two different categories of theoretical approach were reported in both of the relevant categories. Only two trials [59, 60] considered more than one dimension of loneliness (social and emotional/intimate loneliness) but only one of these trials described a ToC that considered each dimension separately [60]. Two other trials (comparing three groups) included a ToC for only one of their evaluated interventions [55, 63].

Mode of intervention delivery was primarily face-to-face, but ten interventions were digital (via video conferencing, telephone calls, mobile texts, a mobile app, or internet-based modules) [49, 50, 52, 54, 57, 58, 63, 64] including two that were blended (face-to-face and digital) interventions [49, 50]. Intervention length varied from four days [63] to two years [46]. Duration of follow-up varied from immediately after treatment completion to nine months after treatment completion [62]. Seventeen interventions were group interventions, eight were individual interventions, and three were integrated individual and group-based interventions. This latter category applied to the Increasing Social Competence and Social Integration of Older Adults experiencing Loneliness (I-SOCIAL) intervention [48], the low-intensity CBT mHealth-supported intervention combined with a group psychoeducation component on technology acceptance [49], and an educational programme combined with individual telephone-based support [65] (see Supplementary Table 2).

### Study quality

No studies were rated as having a low overall risk of bias, twelve studies were rated as having some concerns, and ten were rated as having a high risk of bias (see Supplementary Table 3).

### Study findings

#### Results of individual studies

We report the findings of studies grouped by theoretical approach, with the basis for each theoretical approach summarised in Appendix 3 (see Additional File 3: Appendix 3). Full details of study design are provided in Supplementary Table 1 (see Supplementary Table 1), details of ToC in Supplementary Table 2 (see Supplementary Table 2), and details of quality assessment in Supplementary Table 3 (see Supplementary Table 3).

#### Cognitive-behavioural interventions

Ten studies evaluated cognitive-behavioural interventions.

### Theory of change

The ToC outlined in the included evaluations of CBT interventions were: i) cognitive reappraisal of maladaptive cognitions and reframing perceptions of loneliness, ii) addressing psychosocial barriers that maintain loneliness; iii) reducing avoidance behaviours and promoting social engagement, and iv) developing adaptive coping skills. The final common pathway for such changes was a break in the cycle of behaviours and thoughts that maintain loneliness, which would then increase factors such as value placed on social connections, reductions in negative emotions in social interactions and improvements in self-esteem and self-efficacy (see Supplementary Table 2).

### Individual study findings

A U.S trial by Conoley and Garber [55] compared the effects of two cognitive-behavioural interventions (a reframing intervention and a self-control intervention) addressing attributional styles regarding loneliness. When both were compared to a waiting list control group, all three groups reported lower loneliness scores over time, but with no group differences. Neither intervention was therefore found to be effective in reducing loneliness. The study was judged to be at high risk of bias, all 57 participants were female students, and no power calculation was reported.

A U.S trial by McWhirter and Horan [60] evaluated the effects of two cognitive-behavioural interventions, each designed to address one of two dimensions of loneliness (intimate and social) by addressing attributional styles. The trial used a small sample (*n* = 49) of students and found that participants in the social loneliness intervention group reported decreased intimate and social loneliness. The intimate loneliness intervention was not associated with any significant changes on any of the three loneliness scales. However, it was unclear if group differences remained significant after correction for multiple testing. The study was judged to be at high risk of bias, with no priori power calculation reported, and one of the three loneliness measures used was unvalidated.

A U.S trial by Theeke et al. [53] evaluated a group intervention focusing on rethinking the experience of loneliness (LISTEN), involving group sessions providing educational information on ageing with 27 participants. The LISTEN group reported significantly less loneliness (*p* = 0.018) than the attention control group. The study was judged to be at high risk of bias, no power calculation was reported, and 24 of the 27 participants were female.

An Israeli trial by Cohen-Mansfield et al. [48] evaluated a CBT-informed individual and group intervention for older adults. The trial found a significant reduction in loneliness at three-month post-intervention follow-up (*d* = 0.24, *p* < 0.05) compared to treatment as usual. However, the study was judged to be at high risk of bias, 80% of participants were female, and no sample size calculation was reported.

A South African trial by Jarvis, Padmanabhanunni, and Chipps [49] evaluated a CBT-informed intervention for older low-income adults. No significant differences in loneliness were found immediately at the end of treatment, but a statistically significant reduction in loneliness scores in the intervention group at one-month post-treatment (*X*^*2*^ = 14.62, *p* = 0.001). However, some concerns were identified regarding risk of bias and a small sample size (*n* = 32).

A U.S study by O’Day et al. [62] conducted a three-arm trial comparing CBT to mindfulness-based stress reduction (MBSR) and a waitlist control group. This study found that CBT led to reduction of loneliness post treatment (CBT vs waitlist: *d* = 0.29, *p* = 0.004). However, there were no significant differences between CBT and MBSR post-treatment (*d* = 0.05, *p* = 0.60), and no significant changes at follow up (3, 6, 9, and 12 months later) in loneliness (*p*s > 0.05). This study was judged to be at high risk of bias, particularly relating to measurement of outcomes and generalisability of findings attributed to gender bias and a focus on individuals seeking treatment.

A Swedish trial by Käll et al. [57] evaluated an internet-based CBT (ICBT) intervention for adults experiencing frequent loneliness. There was a statistically significant mean difference in UCLA Loneliness Scale scores between trial arms (intervention and waiting list control) at end of treatment follow-up (*d* = 0.77, *p* = 0.003). The study raised some concerns about risk of bias, with measurement of outcome and selection of the reported results rated as being at moderate risk of bias. Käll et al. [58] also conducted a replication and extension of the trial and used a three-arm RCT evaluating a therapist guided internet-based CBT intervention to an internet-based interpersonal psychotherapy. The ICBT programme yielded significant reductions in loneliness compared to the waitlist with effect sizes that were moderate to large (*d* = 0.71, *p* = 0.006); and moderate compared to internet-based interpersonal psychotherapy (*d* = 0.53, *p* = 0.012). However, there are some concerns regarding risk of bias including deviations from intended treatment, measurement of outcome and selection of reported results.

A U.S trial by Breuhlman-Senecal et al. [64] evaluated an app (Nod) delivering cognitive and behavioural skill-building exercises to college students. The pilot trial found the app to be no more effective than a waiting list control in reducing loneliness (*F* (1, 211) = 0.05, *p* = 0.82; ηp^2^ < 0.001). This study carried some concerns about risk of bias relating to the short period of follow-up and the lack of engagement with the intervention by the young people involved in the trial.

An Israeli trial by Shapira et al. [52] evaluated a CBT-based digital group intervention delivered to older adults via Zoom. The pilot trial found a significant improvement in the intervention group compared to a waiting list control, with a medium effect size identified using mixed effect models (*d* = 0.58). This study was rated as having some concerns about risk of bias relating to the sample size and the use of an unequal allocation to each study arm.

### Summary of findings

This was the only category for which it was feasible to conduct meta-analysis, given the number of studies and homogeneity of measures. This was based on the findings of seven [48, 49, 52, 57, 58, 62, 64] of the 10 CBT studies in this category: one study was excluded on the basis that it had analysed data on six different loneliness outcome measures without specifying which was the primary outcome [60], two studies were excluded (from the main meta-analysis because their authors did not respond to our query about missing information on the number of participants at the outcome measurement time-point [53, 55].

**Fig. 2:**
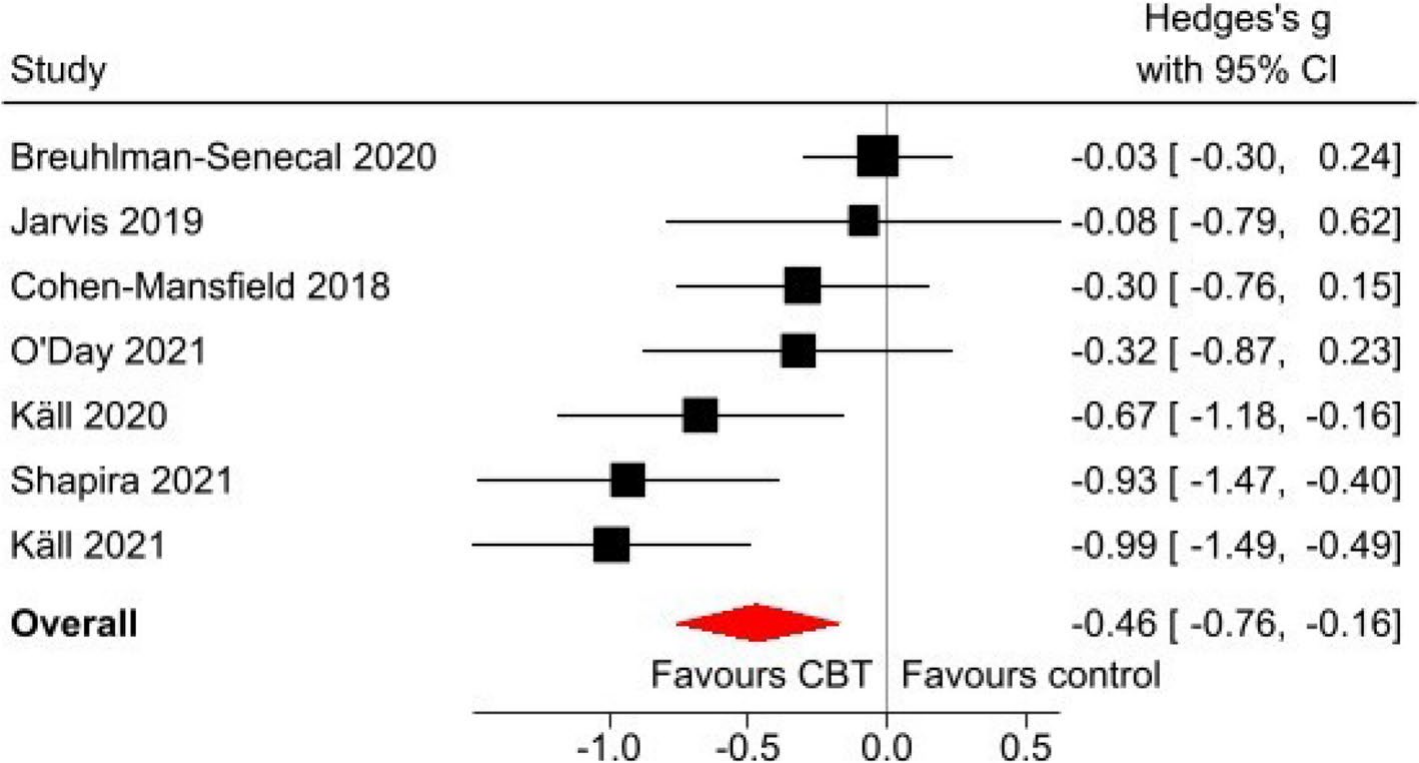
Forest plot

We estimated a pooled effect size of *g* = −0.463 (95% CI: [−0.765 to −0.162]), indicating that the CBT interventions were associated with a moderate reduction in loneliness [66]. However, effect sizes ranged from −0.03 to −0.99 across studies (see Fig. 2). The I^2^ statistic was 64% indicating that substantial proportion of the total variability in effect sizes was due to between-study heterogeneity. A funnel plot was visually examined and demonstrated no evidence of publication bias. The pooled effect size for the sensitivity analysis (−0.430 [95% CI: −0.683 to −0.176]) was marginally smaller than that for the main analysis.

In summary, eight of the ten trials evaluating cognitive-behavioural interventions reported that at least one of the interventions evaluated in each trial provided evidence of effectiveness in reducing loneliness (see Supplementary Table 1). Meta-analysis identified a moderate effect size overall, whilst acknowledging some concerns about risk of bias.

#### Mindfulness

Although there is no specific mindfulness theoretical model of loneliness, we identified four evaluations of mindfulness-based approaches used to improve social engagement and address loneliness.

### Theory of change

The ToC underpinning interventions based on mindfulness intended to change a person’s relationship to maladaptive thoughts linked to loneliness by using techniques (e.g. meditation, mindful yoga, concentration) to increase mindful awareness and acceptance of these thoughts, and improve emotional regulation, self-compassion and self-care, and increase self-efficacy (see Supplementary Table 2). This in turn might reduce negative affect and distress, enhance social functioning and the ability to disengage from perceived social threats, thus reducing loneliness [67].

### Individual study findings

All four trials in this category involved face-to-face group-based interventions, one of which also evaluated CBT as a comparator intervention [62]. A U.S study by Creswell et al. [68] evaluated a mindfulness-based stress reduction (MBSR) intervention with 40 older adults, randomised to MBSR or a waiting list control group. This study found that those in the MBSR group reported a significant reduction in loneliness whilst those in the control group increased slightly, with statistically significant group difference post-treatment (*F* (1,35) = 7.86, *p* = 0.008). Some concerns were identified when assessing risk of bias regarding the selection of the reported results, and no power calculation was reported.

A Chinese trial by Zhang et al. [61] evaluated a face-to-face mindfulness-based cognitive therapy. The pilot RCT randomised 50 university students identifying as lonely, aged 17 to 25 years, to either mindfulness-based cognitive therapy or a waiting list control group. The trial found that the mindfulness training group showed a greater degree of baseline to post-test reduction in loneliness (measured using the validated Chinese loneliness scale) compared with the control group, with a significant time x group interaction effect (*F* (1, 41) = 5.10, *p* = 0.03). Despite randomisation, participants in the intervention group tended to be older and included more graduate-level students relative to the control group, but the analyses were adjusted for age and gender. The study was judged to be at high risk of bias and no power calculation was reported.

A U.S trial by O’Day et al. [62] evaluated a group-based face-to-face intervention that promotes the practice of meditation and mindfulness, and acceptance strategies to encourage acceptance of negative thoughts and anxiety. This study found that MBSR led to reduction of loneliness post treatment (MBST vs waitlist: *d* = 0.34, *p* = 0.001). There were no significant differences observed between CBT and MBST post-treatment, with a small effect size (*d* = 0.05, p = 0.60). This study was judged to be at high risk of bias particularly relating to measurement of outcomes.

A South Asian multi-centre trial by Pandya [46] evaluated a group meditation program. This was trialled in retired South Asian adults in their 60s who were randomised to a waiting list control or to a weekly group meditation program over two years in four cities in India, Nepal, Burma and Sri Lanka, supplemented by home practice [46]. A significant difference in loneliness scores was reported between the two groups at the end of the programme, measured using the de Jong Gierveld Loneliness Scale. However, due to issues with clarifying outcome measurement, we were unable to infer evidence of effectiveness as it appeared that those in the intervention group had become lonelier.

### Summary of findings

In summary, three of the studies in this category found that mindfulness-based interventions were effective at reducing loneliness in adults aged over 55 [68], adults who met criteria for social anxiety [62], and students aged 17–25 years [61] (see Supplementary Table 1). Each had methodological problems (see Supplementary Table 3). One study did not report a power calculation [68], and the others had concerns related to measurement of outcome [62] and no power calculations [61]. A third study evaluating group meditation for retired adults provided inconclusive evidence regarding this approach due to methodological concerns [46]. Our GRADE rating suggested that there is evidence of moderate certainty supporting the effectiveness of interventions underpinned by mindfulness in reducing loneliness (see Additional File 4: Appendix 4).

#### Social identity theory

One trial evaluated an intervention based on social identity theory.

### Theory of change

The ToC underpinning the one intervention evaluated based on social identity theory focused on building one’s sense of identity, agency and purpose through promoting supportive group membership and identification, and a sense of belonging which would then improve self-esteem and reduce psychological distress, therefore reducing loneliness (see Supplementary Table 2).

### Individual study findings

Only one eligible study in this review [56] evaluated an intervention based on social identity theory, Groups 4 Health, trialled in Australia (see Supplementary Table 1). The trial randomised adults with a range of diagnosed mental health conditions or self-reported symptoms of depression to Groups 4 Health or a treatment as usual control group [56]. At the end of treatment, the intervention group reported a greater reduction in loneliness than controls (*d* = − 1.04), measured using the UCLA Loneliness Scale. Attrition was 36% in the intervention group and 24% in the control group. The study reported a power calculation but was assessed as having some concerns about bias based on the highly variable nature of the control group in their receipt of antidepressant medication or psychological therapy, as well as some questions about the fidelity of the intervention itself.

### Summary of findings

In summary, the one study evaluating an intervention based on social identity theory, which was among those studies judged to have the fewest methodological concerns (see Supplementary Table 3), provided medium strength evidence to support the effectiveness of the Groups 4 Health intervention in reducing loneliness in adults with pre-existing mental health problems (see Supplementary Table 1).

#### Self-management of well-being theory

One trial evaluated an intervention based on Self-Management of Well-being Theory (SWB).

### Theory of change

The ToC underpinning this one intervention based on SWB intended to improve social integration and embeddedness by improving one’s ability to attend to and fulfil one’s own needs, to achieve and maintain valued social relationships and social resources. It also aimed to reframe negative thoughts into positive thoughts. Together, through these pathways, the intention was that this would then reduce emotional and social loneliness (see Supplementary Table 2).

### Individual study findings

The one eligible study in this review aimed to improve self-management skills. This Dutch trial compared the effectiveness of a self-management group to that of a control group, the content of which was not described, by randomising 142 single women aged 55 years and above [59]. Although a decrease in social and emotional loneliness was reported in both groups there were no apparent group differences at follow-up, measured using the De Jong Gierveld Loneliness Scale, although no formal statistical tests were provided for group comparisons beyond statistical tests describing reductions in loneliness in each group. Therefore, we cannot ascertain the effectiveness of this intervention. This study was judged to be at high risk of bias, no power calculation was reported, and no group comparisons of the end outcome were provided (see Supplementary Table 3).

### Summary of findings

In summary, the one study evaluating an intervention based on SWB theory did not provide any evidence to support its use in reducing loneliness and this remains an evidence gap (see Supplementary Table 1).

#### Behavioural activation

One trial evaluated an intervention based on behavioural activation.

### Theory of change

The ToC targeted in behavioural activation interventions included addressing avoidance strategies through activity scheduling and modifiable behaviours to promote active social engagement and opportunities that would be rewarding and meaningful; therefore, promoting positive emotions, a sense of connection and closeness and ultimately reducing loneliness (see Supplementary Table 2).

### Individual study findings

Only one eligible study evaluated a digital intervention trialled in a U.S. study [54]. It used a behavioural approach to increase and reinforce wellness-promoting behaviours (e.g., engaging in meaningful life activities aligned with personal values) and to decrease depressive behaviours. Participants, who were housebound older adults, were randomised to a lay-coach-facilitated, video-conferenced, short-term behavioural activation (Tele-BA) intervention or an active control group, who received a weekly video-conferenced Friendly visit (Tele-FV). At six weeks and twelve weeks after the intervention, Choi et al. [54] found that Tele-BA participants reported successively lower loneliness scores, and group comparisons indicated that over this period, the intervention group had higher levels of satisfaction with social support (*t* (82) = 2.00, *p* = 0.049) and lower levels of loneliness (*t* (81) = −3.08, *p* = 0.003) than the control group, with effect sizes for reducing loneliness judged to be medium. This study was judged to have some minor concerns about bias, based on the statistical presentation of group comparisons.

### Summary of findings

In summary, the one study evaluating behavioural activation in this review represented weak evidence to support the use of a video-conferenced, short-term behavioural activation (Tele-BA) intervention in housebound older adults in the U.S. (see Supplementary Table 1). This study was judged to have some concerns regarding risk of bias [54] (see Supplementary Table 3).

#### Reminiscence therapy

Three trials evaluated interventions based on reminiscence therapy.

### Theory of change

Interventions based on reminiscence therapy were based on a ToC that focused on improving cognitive functioning, strengthening one’s sense of self-identity and belonging, finding meaning in life, and a sense of cohesion and acceptance between people with shared experiences to improve mood and psychological wellbeing and reduce and ease the pain of loneliness (see Supplementary Table 2).

### Individual study findings

Three studies in this review evaluated group interventions based on reminiscence therapy for older people. Chiang et al. [47] compared reminiscence therapy for older people in a Taiwanese nursing home to waiting list control and found that at three-month post intervention follow-up loneliness was reduced significantly compared to those in the control group (*z* = 122.75, *p* < 0.0001). However, the study was judged to be at high risk of bias and did not report a sample size calculation.

Ren et al. [51] compared reminiscence therapy to routine community health education in adults aged over 60 and found that at one month follow-up loneliness scores decreased significantly (*t* = 2.01, *p* = 0.001) compared to the control group (*t* = 0.047, *p* = 0.05). This study was judged to be at high risk of bias due to concerns regarding the randomisation process, potential deviations from intended intervention, measurement of outcome and selection of the reported outcomes.

Li et al. [50] evaluated reminiscence therapy in adults aged over 60 living alone and found significant reductions in loneliness both immediately following the intervention and at three-month (T1) and eight-month (T2) follow-up compared to the control group (*M* = 47.43, *SD* = 1.12 and *M* = 46.10, *SD* = 1.04, respectively). Post-test analysis at T1 revealed a significant primary effect for the group factor on loneliness (*F* = 24.133, *p* < 0.001), and a significant primary effect of time on loneliness (*F* = 46.202, *p* < 0.001), indicating improvements in loneliness persisted over the course of the study. This study carried some concerns about risk of bias related to potential deviations from intended intervention and the limited sample size (*n* = 60).

### Summary of findings

In summary, one study was judged to have some concerns regarding risk of bias [50], and the other two were judged to have high risk of bias [47, 51] (see Supplementary Table 3). Overall, our GRADE rating suggested that there is evidence of moderate certainty supporting the effectiveness of interventions based on reminiscence therapy in reducing loneliness (See Additional File 4: Appendix 4).

#### Interpersonal psychotherapy

One trial evaluated an intervention based on interpersonal psychotherapy.

### Theory of change

The interpersonal psychotherapy intervention evaluated here was based on a ToC that aimed to target maladaptive cognitive patterns that result from significant interpersonal events such as grief and interpersonal conflict, based on the idea that this may contribute to the onset of loneliness (see Supplementary Table 2).

### Individual study findings

Only one Swedish study by Käll et al. [58] evaluated a therapist-guided intervention based on interpersonal psychotherapy (see Supplementary Table 1). This internet-based intervention was delivered to adults experiencing distress resulting from loneliness. The study found that the interpersonal psychotherapy group did not exhibit a statistically significant decrease in loneliness during the study duration compared to the ICBT group (*b* = –3.87, 99% CI [–7.28 to –0.45], *SE* = 1.32; *p* = 0.012; *d* = 0.53) or the wait list control group (*b* = –1.36, 99% CI [–4.19 to 1.48], *SE* = 1.44, *p* = 1; *d* = 0.18). This study was judged to have some concerns in relation to deviations from intended treatment and measurement of outcome, alongside concerns regarding generalisation as the majority of the sample were women with university degrees (see Supplementary Table 3).

### Summary of findings

In summary, the one study evaluating interpersonal psychotherapy found no evidence supporting its use in reducing loneliness (see Supplementary Table 1).

#### Orem’s self-care deficits theory

One trial evaluated an intervention based on Orem’s self-care theory.

### Theory of change

The ToC underpinning the intervention, based on Orem’s self-care theory, intended to increase the performance of self-care activities among people with physical illnesses to maintain psychological and physical wellbeing. It was also intended to improve social relationships by setting goals towards maintaining social interaction which would then promote self-agency, sufficiency, and empowerment that then reduce feelings of loneliness (see Supplementary Table 2).

### Individual study findings

Only one eligible study in this review evaluated an intervention based on Orem’s self-care deficit theory [65] (see Supplementary Table 1). This Turkish group-based programme was delivered to people diagnosed with colorectal cancer receiving chemotherapy to promote patient self-care agency. The study found significant statistical differences between the mean loneliness scores for the intervention group and the control groups (*F* = 74.50, *p* < 0.001). The intervention group’s mean loneliness scores were significantly lower compared to the control group at time 1 (the first chemotherapy treatment) and time 2 (the third chemotherapy treatment) (*t* = − 5.081, *p* < 0.001; *t* = − 7.098, *p* < 0.001, respectively). This study carried some concerns related to measurement of outcome, selection of reported results, small sample size, and researchers were not blinded to group assignment (see Supplementary Table 3).

### Summary of findings

In summary, the one study evaluating a self-care intervention based on Orem’s self-care deficit theory provides medium strength evidence supporting the effectiveness of self-care interventions in reducing loneliness among people with colorectal cancer (see Supplementary Table 1).

#### Imagined interaction theory

One trial evaluated an intervention based on imagined interaction theory.

### Theory of change

The ToC underpinning the intervention based on Imagined Interaction Theory was focused on promoting awareness and cognitive and attentional processing of previously avoided painful memories and preparing people to confront potential triggers in social interactions which would then improve psychological outcomes including loneliness (see Supplementary Table 2).

### Individual study findings

One eligible study in this review evaluated an intervention using the expressive writing paradigm for Imagined Interaction [63]. The analysis did not find any significant impacts of time (*F* = 1.75, η^2^ = 0.013) or group by time interaction effects (*F* = 0.49, η^2^ = 0.008) on loneliness. This study was judged to have some concerns in terms of risk of bias, especially regarding the randomisation process and the selection of the reported results (see Supplementary Table 3).

### Summary of findings

In summary, the one study evaluating an intervention based on Imagined Interaction Theory did not provide evidence to support the effectiveness of replay and rehearsal expressive writing tasks in reducing loneliness (see Supplementary Table 1).

#### Logotherapy

One trial evaluated an intervention based on logotherapy.

### Theory of change

The logotherapy intervention evaluated here was based on a ToC that focused on promoting a sense of purpose and meaning in life by identifying values and setting goals, fostering a sense of shared understanding and meaning between people thereby reducing existential loneliness (see Supplementary Table 2).

### Individual study findings

The one eligible study in this review delivered ten sessions of logotherapy to Iranian patients with advanced-stage cancer [69] (See Supplementary Table 1). The study found a statistically significant difference in the mean existential loneliness scores for the intervention and control group, with the logotherapy group presenting lower scores (*t* (61) = −5.19, *p* < 0.001). Furthermore, compared to pre-test scores, post-test existential loneliness scores were significantly lower in the logotherapy group (*t* (30) = −8.79, *p* < 0.001), while they were significantly higher in the control group (*t* (31) = 3.65, *p* < 0.001). This study raised some concerns about bias related to the selection of the reported results and to the blinding of the researcher to group assignment (see Supplementary Table 3).

### Summary of findings

In summary, the one study evaluating a logotherapy intervention provided evidence of medium strength supporting the effectiveness of group logotherapy in reducing existential loneliness among people with cancer at an advanced stage (see Supplementary Table 1). This study raised some concerns about bias related to the selection of the reported results and to the blinding of the researcher to group assignment (see Supplementary Table 3).

#### Cost effectiveness evaluations

None of the included studies assessed cost-effectiveness.

## Discussion

### Main findings

This systematic review of 22 trials distils the ToC and the evidence describing effectiveness for 29 interventions designed to reduce loneliness using psychological strategies and confirmed an absence of cost-effectiveness data. Its novelty lies in presenting evidence for effectiveness alongside details of the underlying ToC to clarify how and/or why an intervention might have worked [22]. Some ToCs were very clear and some were less well elaborated (particularly in the category of Mindfulness) although identifiable through the information on intended pathways provided by the authors. Clarity of ToC was similar for whether interventions were digital, non-digital or blended. Only one trial used a ToC that considered two separate ToCs, each addressing different dimensions of loneliness separately [60]. This provided only weak evidence to support one of the interventions trialled, but separating out dimensions of loneliness in this way was helpful mechanistically. The detailed work we conducted in identifying these theoretical processes and likely mechanisms provides a useful resource for those interested in adapting individual interventions supported by effectiveness data for other age groups, clinical conditions, or cultural settings.

The 22 eligible studies including loneliness as a primary outcome covered six categories of psychological approaches. These trials presented evidence to support the effectiveness of eight interventions evaluating *cognitive-behavioural* approaches (of 12 trialled in total across 10 studies), three interventions evaluating *mindfulness-based* approaches (of four trialled in total across three studies), one intervention based on *social identity theory* (of one trialled), one intervention based on *behavioural activation* (of one trialled), three interventions based on *reminiscence therapy* (of three trialled), one intervention based on *Orem’s self-care theory* (of one trialled), and one intervention based on *logotherapy* (of one trialled).

The trials did not find evidence to support the effectiveness of: 1) two *cognitive-behavioural* interventions (of 12 trialled in total across 10 studies), one of which was a pilot trial [64], and the other a three-group RCT [55],2) one *mindfulness* intervention (of four trialled in total across three studies) [46],3) one intervention based on *SWB theory* (of one trialled) [59], 4) one *interpersonal psychotherapy* intervention [58], and 5) an *imagined interaction* intervention [63].

All the above findings must be interpreted in the context of a medium to high risk of bias, with ten studies rated as being at high risk of bias, mainly due to small sample sizes, lack of an a priori power calculation, unvalidated measures, short duration of follow-up, and selective outcome reporting. Of the total 22 eligible studies, 17 studies included more than one primary outcome capturing loneliness, increasing the potential for Type I error. These methodological problems reduce the validity of respective findings, and the degree of confidence in implementing those interventions found to be effective. The absence of cost-effectiveness studies also makes it hard for policymakers to consider allocative efficiency when resourcing psychological services.

In summary, taking into account methodological limitations, we identified medium strength evidence to support i) eight *cognitive-behavioural* interventions, ii) one intervention based on *social identity theory* (Groups 4 Health), iii) three interventions using *reminiscence therapy*, iv) one intervention based on *Orem’s self-care deficit theory* and v) one intervention based on *logotherapy*. We identified very weak evidence to support i) one intervention based on *behavioural activation* and ii) three *mindfulness* interventions. We found no evidence to support an intervention based on *SWB theory*, another based on *interpersonal psychotherapy*, and one based on *imagined interaction theory*.

We noted that studies in which the interventions’ ToC was described with clarity and sufficient level of detail were also those published more recently (with the exception of [68]). The most consistent evidence supported the use of cognitive-behavioural approaches to address loneliness. However, as the most well-established approach, these represented the majority of interventions evaluated in this review. These were also the interventions setting out the most coherent ToC. However, small sample sizes and limited follow-up in this category suggest a need for larger trials with longer follow-up to assess whether reductions in loneliness persist, and trialling in other settings to test consistency. Of note, longer-term outcomes have since been published for one of the pilot trials included [57] but that study was ineligible for inclusion because both study arms had received the active intervention [70]. The study found further significant reductions in loneliness scores for the original intervention, suggesting sustained benefits [70].

### Strengths and limitations

A key strength of this review lay in setting out the ToC for each intervention included, clarifying the mechanisms of therapeutic shifts (how modification of thoughts and feelings relating to loneliness was approached) in those interventions shown to be effective. Our review focussed specifically on RCTs, the gold standard in testing effectiveness [71], that specified loneliness as a primary outcome, to ensure that their primary aim was to reduce loneliness. Our strict inclusion criteria will have omitted trials of a number of other interventions designed to treat physical or mental health problems (or transdiagnostic processes such as shame) in which loneliness was not the focus, but where social connectedness was promoted through group formats or where cognitions influencing loneliness were modified. However, we felt it was important to focus on this sub-group of defined trials rather than broadening out our summary of such literature to include trials without the primary aim of addressing loneliness. This is because evaluations of loneliness as a secondary outcome would provide far less clarity over precise mechanisms of reducing loneliness [31, 72]. This is where our review makes a distinctive contribution to the literature and to intervention development. Our review was also intentionally narrow in that it was restricted to studies sampling participants without intellectual impairments or dementia because the psychological interventions for these groups are more specific [38, 39]. Our findings are therefore not generalisable to groups with cognitive impairment. Additionally, there may be a risk of publication bias, particularly given that we did not search for grey literature (e.g. thesis, preprints).

### Results in the context of other studies

Our findings regarding the effectiveness of psychosocial interventions addressing loneliness are complemented by those of a recent systematic review focused on identifying the active components of social, educational or psychological interventions found to be effective in addressing loneliness in the general population [73]. In terms of the psychological interventions, the authors identified one CBT study, one intergenerational reminiscence intervention, and one MBST intervention study that were effective at reducing loneliness, and one CBT study that utilised active participation methods with unclear effectiveness. Similar to our findings, Morrish, Choudhury, and Medina-Lara [73] emphasised that CBT was an effective learning mechanism and that the one effective intervention identified in their review that utilised CBT principles showed significant reduction in loneliness. This review was aimed at extracting the “themes, structures, and tasks” and content used in each intervention [73]. The authors characterised five features of effective interventions: 1) use of in-between session interaction, 2) active participation, 3) opportunities for group/facilitator interaction, 4) variation in teaching and learning styles, and 5) inclusion of a clear learning mechanism [73]. These are important findings for considering the delivery of the interventions found to be effective in our systematic review.

Our findings are also consistent with those of an umbrella review of systematic reviews on the effectiveness of interventions targeting loneliness in older persons, in which among a heterogenous range of interventions trialled, there was strongest evidence for a social cognition intervention [74]. This systematic review also found that interventions with a clear theoretical basis, usually behavioural change theory, were found to be more effective compared to those lacking theoretical underpinnings [74]. Another published systematic review and meta-analysis of studies trialling psychological interventions targeting loneliness presented findings complementing our review although was broader in remit (including trials based on a psychological theory, but not all specifying their ToC) [31]. The broader range of interventions evaluated included integrative approaches, social skills training, interpersonal therapy, and a gratitude intervention, as well as those based on cognitive-behavioural therapy, mindfulness-based approaches, social identity theory and reminiscence therapy [31]. As with our review, the most common approaches evaluated were cognitive-behavioural. Meta-analysis described a small to medium effect size for psychological interventions in reducing loneliness compared to control group, with no evidence to support the influence of any specific type of psychological intervention [31]. This, the absence of a clear ToC in some interventions trialled, and the considerable heterogeneity in the effectiveness of the interventions, suggests a need for further trials that will elucidate specific mechanisms of change [31].

The literature summarised in our review, as with other reviews, illustrates the increasing investment over the last few decades in RCTs of interventions addressing loneliness, as delivered to older adults [34, 35], young people [27, 36] and adults [24, 25]. Whilst there is no clear agreement on whether social or psychological (or a combination of both) interventions are superior in addressing loneliness [27, 32, 75, 76], our very focused review of psychological interventions has been able to identify promising approaches within this category in more detail.

### Clinical, research and policy implications

On the basis of the evidence summarised in our review, and the explanations provided in each study for the underlying ToC, we feel there are grounds for further work developing interventions founded on five approaches: *cognitive-behavioural therapy, social identity theory, reminiscence therapy, Orem’s self-care deficit theory,* and *logotherapy*. This is based on the medium strength evidence to support the interventions based on these theoretical approaches and the coherence of their underlying theories of change. However, for all approaches trialled, there is a need for methodologically rigorous trials to test replicability of findings in other settings, involving independent research groups, and to improve on the identified methodological problems. Further longitudinal work is also needed to understand mechanisms of change in more detail, based on differing theoretical principles underlying the interventions. Additionally, future interventional studies should consider investigating the effects of interventions on the intended mechanism(s) of change they are designed to address. We should also investigate important social approaches to intervening with loneliness [36, 76] and the mechanisms by which these might work in combination with the psychological approaches analysed in this paper. We noted that only one trial was of multi-component intervention, combining a community-based approach with a psychological approach [48]. Further work is needed to clarify the contribution of psychological interventions within the broader context of other interventions to support people experiencing chronic loneliness and the possible advantages of different elements and multi-format interventions [73]. Such work should also take into account the societal and community context in which participants live, such as access to transport, green space, and opportunities to connect with other [24].

We noted that only five studies trialled interventions in clinical populations of people with mental health problems, specifically those who were depressed [51, 55, 56, 63], and socially anxious [62]. Given that there is a higher risk of loneliness for people with mental health problems than the general population [77], there is a need for further trials in populations of people with a range of mental health disorders. Another research priority is the incorporation of cost-effectiveness evaluations into future trials, as these are noted to have been lacking in the field of loneliness research [14, 15]. This will help policymakers in allocation decisions when commissioning services [78], particularly when balancing the economic cost of loneliness and social isolation [15], the potential cost savings in reducing loneliness [78], and restrictions on funding for mental health services. The majority of cost-effectiveness evaluations of interventions targeting loneliness have been conducted in samples of older adults in the UK [15] and this needs rebalancing, particularly given the likely long-term effects of loneliness in younger people.

## Conclusions

Knowledge about the psychological factors that contribute to loneliness is increasing rapidly. However, research on interventions targeting psychological factors to reduce loneliness is still in development. Our review identified 22 studies evaluating psychological interventions to reduce loneliness that were based on a discernible ToC. Our synthesis presented grounds for further work developing interventions founded in cognitive-behavioural approaches, social identity theory, reminiscence therapy, Orem’s self-care deficit theory, and logotherapy, based on medium strength evidence and underlying ToC coherence. Future research should focus on developing theoretically-informed psychological interventions to reduce loneliness in specific groups and settings, then conducting feasibility/pilot studies followed by fully-powered methodologically rigorous RCTs incorporating cost-effectiveness evaluations.

## Supplementary Information

Additional file 1: Appendix 1. PRISMA checklist for systematic reviews.

Additional file 2: Appendix 2. PRISMA checklist for abstracts.

Additional file 3: Appendix 3. Summary of theoretical approaches for each intervention category.

Additional file 4: Appendix 4. GRADE scoring criteria for interventional studies investigating the effectiveness and cost-effectiveness of interventions intended to reduce loneliness using psychological strategies and a theory of change.

Additional file 5: Supplementary Table 1. Characteristics of included studies classified by intervention type: *n *= 22.

Additional file 6: Supplementary Table 2. Details of interventions and their theory of change classified by intervention type.

Additional file 7: Supplementary Table 3. Risk of bias in included studies classified by intervention type.

## Data Availability

All data supporting the conclusions of this article are published and in the public domain. The datasets used and/or analysed during the current study are available from the corresponding author on reasonable request.

## Abbreviations

ToC: Theory of change
CBT: Cognitive behavioural therapy
MBSR: Mindfulness-based stress reduction
MRC: Medical research council
SWB: Self-management of well-being
SD: Standard deviation
N: Number of participants
