# Supplementary material for: Investigating the effectiveness of interventions intended to reduce loneliness using psychological strategies and a theory of change: a systematic review of interventional studies and meta-analysis
Source: BMC Psychol. 2025 Dec 12;14:131. doi: 10.1186/s40359-025-03639-3 (PMC12857015; doi:10.1186/s40359-025-03639-3)
Supplement: Supplementary file 3 — Additional file 3: Appendix 3. Summary of theoretical approaches for each intervention category. [file 40359_2025_3639_MOESM3_ESM.docx]

**Additional File 3**

**Appendix 3: Summary of theoretical approaches for each intervention category**

*Cognitive behavioural approaches*

Cognitive-behavioural theory posits that an individual’s cognitions, and specifically their core negative assumptions about themselves and others, strongly influence and interact with their emotional and behavioural responses to life situations, and thus the development and maintenance of symptoms of psychopathology (Käll et al., 2021). Cognitive behavioural therapy might involve using exposure-based techniques and/or techniques to re-frame or re-structure maladaptive cognitions that produce and maintain loneliness and limit social interactions.

*Mindfulness-based approaches*

Mindfulness-based approaches focus on purposively monitoring and attending to one’s experiences in the moment with a non-judgmental attitude. Mindfulness-based interventions aim to promote openness and non-judgmental awareness of one’s mind and body (Hofmann & Gómez, 2017). This non-judgmental awareness of thoughts and emotions can help people emotionally regulate and promote self-acceptance and compassion which in turn improves emotional wellbeing. This approach can involve body scanning, meditation and gentle stretching.

*Social identity Theory*

The social identity approach to reducing loneliness predicts that when people gain a sense of social group-based belonging, they are provided with a sense of meaning, connection, support and agency; and this sense of positive identity improves wellbeing and reduces depression and loneliness (Haslam et al., 2022). The theory suggests that such changes in social identity are achieved through helping people to find and sustain the group memberships that would provide these psychological resources.

*Self-management of well-being theory*

The Self-Management of Well-being (SWB) theory postulates that if people have good self-management abilities, namely skills enabling them to achieve and maintain key physical and social resources, they can ensure good well-being. The theory suggests that where applied to achieving and maintaining friends, a key dimension of wellbeing (affection) is satisfied, thereby improving well-being and reducing social and emotional loneliness. These self-management abilities are conceptualised as: 1) taking initiatives; 2) being self-efficacious; 3) investing; 4) having a positive outlook; 5) ensuring multi-functionality in resources; and 6) ensuring variety in resources (Kuiper et al, 2019). Interventions based on SWB theory use psychological approaches that enhance self-management abilities in relation to friendships and are therefore seen to have the potential to reduce loneliness.

##### *Behavioural activation*

Behavioural activation is grounded in learning theory and uses activity scheduling of avoided behaviours and functional analysis of the cognitive processes that involve avoidance, which together enhance change in behaviour towards goals (Janssen et al, 2021).

*Reminiscence therapy*

Reminiscence therapy is intended to improve cognitive functioning (specifically memory) to facilitate social interaction through the recollection and sharing of memories and emotional experiences (Chiang et al., 2021).

*Interpersonal psychotherapy*

Interpersonal psychotherapy is focused on the link between mood and interpersonal interactions/events such as life changes (Käll et al., 2021). It uses a combination of role-play, communication analysis, and experiential exercises (Käll et al., 2021).

*Orem’s self-care deficit theory*

Orem’s self-care deficit theory underpins self-care nursing programmes focused on supporting and enhancing individual’s capacity for self-care, encompassing both the amount and quality of self-care practices (Ökten & Özer et al., 2022; Queirós et al., 2014).

*Imagined Interaction theory*

Imagined interaction theory suggests that interpersonal communication and relationships are founded on intrapersonal communication (Zhang et al., 2023). This includes both rehearsing expected interactions and imagining different endings to past interactions. Expressive writing can be used to mentally prepare for anticipated interactions that relate to one’s own personal trauma linked to mental health conditions (Zhang et al., 2023).

*Logotherapy*

Through increasing motivation for change, logotherapy aims to support people in finding a deeper meaning in their lives (Heidary et al., 2023). When delivered in group format, individuals will be able to share similar experiences and emotions. In this way, a shared meaning can be created, and individuals might feel more connected to each other, and therefore less lonely.

**References**

Chiang, K.J., Chu, H., Chang, H.J., Chung, M.H., Chen, C.H., Chiou, H.Y., & Chou, K.R. (2010). The effects of reminiscence therapy on psychological well-being, depression, and loneliness among the institutionalized aged. *International Journal of Geriatric Psychiatry*, *25*(7), 380-388. <https://doi.org/10.1002/gps.2350>

Haslam, S. A., Haslam, C., Cruwys, T., Jetten, J., Bentley, S. V., Fong, P., & Steffens, N. K. (2022). Social identity makes group-based social connection possible: Implications for loneliness and mental health. *Current Opinion in Psychology*, *43*, 161-165. https://doi.org/10.1016/j.copsyc.2021.07.013

Heidary, M., Heshmati, R., & Hayes, J. (2023). Effect of group logotherapy on anxiety about death and existential loneliness in patients with advanced cancer: A randomized controlled trial. *Cancer Nursing*, *46*(1), E21-E30. https://doi.org/10.1097/ncc.0000000000001086

Hofmann, S. G., & Gómez, A. F. (2017). Mindfulness-based interventions for anxiety and depression. *Psychiatric Clinics*, *40*(4), 739-749. <https://doi.org/10.1016/j.psc.2017.08.008>

Janssen, N. P., Hendriks, G. J., Baranelli, C. T., Lucassen, P., Oude Voshaar, R., Spijker, J., & Huibers, M. J. (2021). How does behavioural activation work? A systematic review of the evidence on potential mediators. *Psychotherapy and Psychosomatics*, *90*(2), 85-93.

<https://doi.org/10.1016/j.ijans.2019.100177>

Käll, A., Bäck, M., Welin, C., Åman, H., Bjerkander, R., Wänman, M., Lindegaard T., Berg M., Moche H., Shafran R., & Andersson, G. (2021). Therapist-guided internet-based treatments for loneliness: A randomized controlled three-arm trial comparing cognitive behavioral therapy and interpersonal psychotherapy. *Psychotherapy and Psychosomatics*, *90*(5), 351-358. https://doi.org/10.1159/000516989

Kuiper, D., Steverink, N., Stewart, R.E., Reijneveld, S.A., Sanderman, R., & Goedendorp, M.M. (2019). Pace and determinants of implementation of the self-management of well-being group intervention: A multilevel observational study. *BMC Health Services Research, 19*, 67. https://doi.org/10.1186/s12913-019-3891-x

Ökten, Ç., & Özer, Z. (2022). Orem’s Theory with Educational Telephone Follow-ups: A Randomized Controlled Trial. *Nursing Science Quarterly*, *35*(4), 444-454. https://doi.org/10.1177/08943184221115126

Queirós, P., Vidinha, T., & Filho, A. (2014). Self-care: Orem´s theoretical contribution to the nursing discipline and profession. *Revista de Enfermagem Referência*, *IV Série*(3), 157–164. <https://doi.org/10.12707/riv14081>

Zhang, W., Jhang, J., & Greenwell, M. R. (2023). Effects of replay and rehearsal expressive writing on mental health: A randomized controlled trial. *Journal of Mental Health*, *32*(3), 582-591. <https://doi.org/10.1080/09638237.2022.2140783>
