# Supplementary material for: Investigating the effectiveness of interventions intended to reduce loneliness using psychological strategies and a theory of change: a systematic review of interventional studies and meta-analysis
Source: BMC Psychol. 2025 Dec 12;14:131. doi: 10.1186/s40359-025-03639-3 (PMC12857015; doi:10.1186/s40359-025-03639-3)
Supplement: Supplementary file 4 — Additional file 4: Appendix 4. GRADE scoring criteria for interventional studies investigating the effectiveness and cost-effectiveness of interventions intended to reduce loneliness using psychological strategies and a theory of change. [file 40359_2025_3639_MOESM4_ESM.docx]

**Additional File 4: Appendix 4: GRADE scoring criteria for interventional studies investigating the effectiveness and cost-effectiveness of interventions intended to reduce loneliness using psychological strategies and a theory of change**

**Table 1**

*GRADE Score for Mindfulness Interventions*

| Intervention: Mindfulness interventions  Result: Moderate certainty  Contributing studies: 4 (see Additional File 4: Table 1) | | |
| --- | --- | --- |
| **Domain** | **Assessment** | **Outcome** |
| Study quality/risk of bias | Serious concerns: 3 studies rated as high risk of bias, 1 rated as with some risk of bias. | Downgraded to moderate certainty. |
| Inconsistency | Borderline concerns: 3 studies showed intervention to be effective, and for one study evidence of effectiveness cannot be inferred from results. | No change. |
| Indirectness | No concerns: Validated measures used by all studies. All studies investigated general population samples or samples with common mental disorders. | No change. |
| Imprecision | Borderline concerns: Sample sizes ranged from 40 to 378; the smallest sample was an RCT, as were the largest two; one study (n=50) was a pilot RCT. | No change |
| Publication bias | Borderline concerns: Only four studies, one with negative results. | No change |

**Table 2**

*GRADE Score for Reminiscence Therapy*

| Intervention: Reminiscence therapy  Result: Moderate certainty  Contributing studies: 3 – (see Additional File 4: Table 1) | | |
| --- | --- | --- |
| **Domain** | **Assessment** | **Outcome** |
| Study quality/risk of bias | Serious concerns:  2 studies rated as high risk of bias, 1 rated as with some risk of bias. | Downgraded to moderate certainty. |
| Inconsistency | No concerns:  All 3 studies showed intervention to be effective. | No change. |
| Indirectness | No concerns: All studies investigated general population samples. | No change. |
| Imprecision | Borderline concerns: One sample size 60, and two >100. | No change. |
| Publication bias | Borderline concerns: Only three studies, no negative findings. | No change. |
