# Supplementary material for: Investigating the effectiveness of interventions intended to reduce loneliness using psychological strategies and a theory of change: a systematic review of interventional studies and meta-analysis
Source: BMC Psychol. 2025 Dec 12;14:131. doi: 10.1186/s40359-025-03639-3 (PMC12857015; doi:10.1186/s40359-025-03639-3)
Supplement: Supplementary file 5 — Additional file 5: Supplementary Table 1. Characteristics of included studies classified by intervention type: n = 22. [file 40359_2025_3639_MOESM5_ESM.docx]

**Supplementary Table 1**

*Characteristics of included studies classified by intervention type: n (total studies) = 22*

| **Study** | **Design*** | **Population sampled** | **Sample size** | **Intervention** | **Comparator** | **Primary outcome** | **Follow-up** | **Results** |
| --- | --- | --- | --- | --- | --- | --- | --- | --- |
| **Cognitive behavioural approaches (*n* total studies=10)** | | | | | | | | |
| Conoley & Garber, 1985  (USA) | RCT (three groups) | Female students with moderate depression and loneliness  Mean age: Not stated  Female: 100% | 57 | Two interventions evaluated:  reframing intervention to increase attributions  of the controllability of loneliness  self-control intervention focused on overcoming feelings of loneliness  Delivered in 2 sessions | Waiting list control | *UCLA 20-item (Roberts, 1980)  (20 items)  (also measured the degree to which loneliness could be  altered by someone using The Causal Dimension Scale (Russell, 1982)  The controllability subscale used in this study, and  measured depression using the Beck Depression Inventory | End of treatment follow-up  (2 weeks)  Post treatment follow-up  (2 weeks) | Participants in all three groups, including the waiting list control group, became less lonely over time, but there were no group differences so neither treatment was judged effective in reducing loneliness. |
| McWhirter & Horan, 1996  (USA) | RCT (four groups) | Lonely students  Mean age: 24.8 years  Female: 52.3% | 49 (44 analysed) | Group sessions focusing on a) intimate loneliness, b) social loneliness, c) intimate and social loneliness | Self-help discussion group | Loneliness assessed using  1) UCLA Intimate – a 10-item subscale of the Revised UCLA Loneliness Scale (R-UCLA) capturing intimate loneliness (representing one factor on factor analysis of the R-UCLA);  2)  The Intimate Loneliness Scale (ILS), a 10 item scale developed for this study  3) The Differential Loneliness Scale: Romantic-Sexual Subscale (DLS  Romantic-Sexual) is one of four DLS subscales | End of treatment follow-up  (6 weeks)  Post treatment Follow-up  (2 months) | The intimate loneliness intervention was not associated with any significant changes on any of the three loneliness scales.  The social loneliness intervention was associated with decreased  feelings of intimate and social loneliness  (*F* (1, 39) = 6.86, *p* = .01). |
| Theeke et al., 2016  (USA) | RCT | Older adults (65+) who were chronically ill and lonely  Mean age: 75 years  Female: 88.9% | 27 | Weekly group sessions focusing on loneliness, belonging and identifying meaning in peoples experience  (3-5 participants per group, meeting weekly for 2 hours over five weeks) | Educational attention control, receiving weekly group meetings providing scripted educational information on aging | *Revised UCLA Loneliness Scale  (20 items)  (also measured Salivary cortisol, usng the Adrenocortex Stress Profile Kit, and DHEA, IL-6, and IL-2 levels, also measured Psychosocial functioning using Geriatric Depression Scale and Katz ADL and IADL instruments, Social support was measured using the MOS Social Support Scale. Quality of life was measured using a visual analogue scale ranging from 0 to 10. | Post treatment Follow-up  (12 weeks) | The LISTEN group demonstrated  significantly less loneliness (*p* = .018) than that of the  attention control group. |
| Cohen-Mansfield et al., 2018  (Israel) | RCT | Older adults (65+) experiencing loneliness  Intervention group:  Mean age: 76.6 years (SD = 6.8)  Female: 31/39  Control group:  Mean age: 79 years (SD = 6.62)  Female: 29/35 | 89 | Increasing SOcial Competence and social Integration of older Adults experiencing Loneliness (I-SOCIAL) intervention: CBT-informed individual and group sessions on social activity (up to seven individual and seven group sessions)  Treatment end: approximately 6 months after baseline | Treatment as usual | Loneliness assessed using three measures: UCLA Loneliness scale – short form (8 items)  Two items replaced.  Frequency of loneliness was measured via “How often would you say  you feel lonely?” Severity of loneliness was measured via “To what extent do you feel  lonely?”  measured on a 5-point scale from “not at all”  (1) to “to a very large extent” | End of treatment (length of treatment unclear but approximately 7 weeks)  Post treatment Follow-up  (3 months) | Significant reduction in loneliness at end of intervention and at 3 month follow-up compared to control group, *p* < .05. Effect size was 0.29 post-intervention and 0.24 at follow-up |
| Jarvis, Padmanabhanunni, & Chipps, 2019  (South Africa) | RCT | Older low income adults (60+) experiencing loneliness and residing in accommodation for low-income adults (aged 60 and above;  Mean age: 74.93 years (SD = 6.41, range = 61-87)  sample were mainly female,  Asian/Indian, widowed, and primary school and lesser-educated. 62.5% participants  had lived in the residences for 19 months or longer. | 32 | low-intensity CBT delivered face-to-face and via a messaging app (WhatsApp)  3 months | Treatment as usual (a generic wellness  program for all low income older adults in the residences).  For ethical reasons the control group also received  the intervention on completion of the trial. | *De Jong Gierveld Loneliness scale  (6 items)  (also measured Social Cognition using Disconnection and Rejection domain of the Young Schema Questionnaire-Short Form (YSQ-SF), Mental Wellbeing using The World Organisation Five Wellbeing Index (WHO-5)) | End of treatment follow-up  (3 months)  Post treatment Follow-up  (1 month) | Significant reduction in loneliness post intervention and was maintained  one month after the active intervention.  (X^2^ =14.62, *p* = .001) |
| Käll et al., 2020  (Sweden) | Pilot RCT | Adults experiencing frequent loneliness  Mean age: 47.2 years  Female: 52% | 73 | Internet accessed Cognitive-Behavioural therapy (ICBT) programme. 8 week online website-based program with 8 modules | Waiting list | UCLA-Loneliness Scale-Version 3  translated into Swedish (20 items) | End of treatment follow-up  (8 weeks) | Statistically significant mean difference between conditions at post-treatment (*b* = -4.65, *SE* = 1.57, *p* = .003, *d* = 0.77, 95% CI [0.22, 1.33]. |
| Breuhlman-Senecal et al., 2020 (USA) | Pilot RCT | College students  Mean age: 18.7 years  Female: 59% | 221 | Nod, a mobile application, designed to deliver cognitive and behavioural skill-building exercises. Access to app for 4 weeks | Waiting list (but all those in the control group then received the app at the end of the trial) | * UCLA Loneliness Scale, 8-item  (also measured Engagement, Anxiety and Depression Symptoms using Generalized Anxiety Disorder Scale (GAD-7) and the 9-item Patient Health Questionnaire (PHQ-9)  Social Anxiety Symptoms measured by 3-item Mini Social Phobia Inventory Sleep Quality as measured by Pittsburgh Sleep Quality Index; and 4 College Adjustment Indicators: Perceived Social Support, Campus Belonging, Social Adjustment to College, Intention to Return  User Experience) | End of treatment (four weeks)  Post treatment follow-up (four weeks post intervention) | No significant overall effects of the Nod app on loneliness at end of treatment in week 4 (*F* (1, 211) = 0.05, *p* =.82; ηp² <.001) despite exceeding the sample size estimated for adequate power.  There was also no significant effect on  campus belonging  *F* value <1.40; *p >*.23 |
| Shapira et al, 2021 (Israel) | Pilot RCT | Community-dwelling older adults aged between 65 and 90 years  Mean age: 72 years  Female: Not stated | 82 | CBT-based digital group intervention delivered via online video conferencing (Zoom)  Delivered twice weekly over 3 and a half weeks (7 sessions in total) | Waiting list | *3-item version of the UCLA Loneliness Scale  (also measured depressive symptoms using The Patient  Health Questionnaire (PHQ-9) | Post treatment follow-up (3.5 weeks) | The between-group difference in  loneliness scores were  *d* = 0.58, indicating a medium effect size of the intervention. |
| Käll et al., 2021 (Sweden) | RCT (3 groups) | Adults experiencing distress due to loneliness  Mean age: 47.5 years  Female: 75.9%  69.4% had a university degree  76% reported their civil status as either single (52.4%), divorced (17.1%), or widowed (6.5%). | 170 | Two Interventions: Internet-based CBT  Internet-based interpersonal therapy (IPT) | Waiting list control | UCLA-Loneliness Scale-Version 3  (also measured depressive symptoms using the Patient Health Questionnaire-9 (PHQ-9), symptoms of social anxiety using Social Interaction Anxiety (SIAS), symptoms of generalised anxiety using Generalised Anxiety Disorder 7-item scale, quality of life measured using Bruusviken Brief Quality of Life Scale (BBQ)) | End of treatment (10 weeks)  Post-treatment follow-up (4 months) | ICBT programme led to significant reductions in loneliness compared to the wait-list control group (Cohen’s *d* = 0.71, *p* = .006) and IIPT programme (*d* = 0.53, *p* = .012). ICBT programme also led to larger reduction in loneliness during treatment period compared to IIPT. There were no significant changes in loneliness between the posttreatment and follow-up assessments for the ICBT. |
| O’Day et al., 2021 (USA) | RCT (3 groups) | Adults seeking treatment, scoring greater than 60 on the Liebowitz Social Anxiety Scale-Self Report (LSAS-SR) and met criteria for DSM-IV generalised Social Anxiety Disorder based on the Anxiety Disorder Interview Schedule for DSM-IV  Intervention group:  Mean age: 34.14 year (SD = 8.05)  Female: 55.6%  White: 50%  Asian: 41.7%  Latino: 5.6%  Multiracial: 2.8%  Single: 55.6%  Married: 33.3%  Living with partner: 8.3%  Control group:  Mean age: 34.06 year (SD = 7.78)  Female: 55.6%  White: 41.7%  Asian: 38.9%  Latino: 2.8%  American Indian: 2.8%  Multiracial: 13.9%  Single: 50.0%  Married: 44.4%  Living with partner: 2.8%  Not reported: 2.8% | 108 | CBT therapy face-to-face group therapy  Twelve weekly 2 hour and half sessions with homework assignments | Waiting list | *UCLA-8 Loneliness Scale (ULS-8)  (also measured social anxiety using *Liebowitz Social Anxiety Scale-Self-Report* (LSAS-SR)) | End of treatment follow-up (3 months)  Post treatment follow-up (6, 9, 12 months) | Loneliness at post-treatment was significantly lower for CBT versus waitlist (*t* (102.48) = 2.96, *p* = .004, *d* = 0.29). There was no significant difference in loneliness between CBT group and mindfulness-based stress reduction at post-treatment (*t* (105.86) = 0.53, *p* = .60, *d* = 0.05). Loneliness did not decrease linearly across the year following treatment. There were no differences between treatments at post-treatment or during follow-up period (*p’s* > .05). |
| **Mindfulness-based approaches (*n* total studies=4)** | | | | | | | | |
| Creswell et al., 2012  (USA) | RCT (two groups) | Healthy adults aged 55+  Mean age: 65 years  Female: 80% | 40 | Mindfulness-based stress reduction programme  8 weekly group sessions, daily home practice and a day-long retreat | Waiting list | *UCLA-R Loneliness Scale  (20 items)  (also measured:  Mindfulness Skills using the 39-item Kentucky Inventory of Mindfulness Skills (KIMS) and Gene expression profiling and pro-inflammatory protein analysis) | End of treatment follow-up  (8 weeks) | Decrease in loneliness  compared to waitlist control  η^2^ = .17; *p* = .02 |
| Zhang et al., 2018  (China) | Pilot RCT | Chinese college students with high loneliness scores aged 17 to 25 years  Mean age: 20 years  Female: 42% | 50 | Mindfulness-based cognitive therapy, over a course of 8 weeks | Waiting list | *Chinese college student loneliness scale (a 16 item scale, reduced from the original 21 item scale – developed for a Chinese student population - following factor analysis)  (also measured trait Mindfulness using Five facets mindfulness  questionnaire – Chinese version) | End of treatment follow-up  (8 weeks)  Post treatment Follow-up  (3 months) | The mindfulness training group showed a greater degree of reduction on loneliness from the baseline to post-test, compared with the control group, with a significant interaction effect of time x group ( *F* (1, 41) = 5.10, *p* = .03). |
| Pandya, 2021  (India, Nepal, Burma, Sri Lanka) | RCT | retired South Asian older adults  age range 62–68 years; retired for 2–5 years at the commencement of the study | 378 | Group meditation program weekly for 2 years supplemented by weekly home practice | Treatment as usual | *De Jong Gierveld Loneliness Scale (six-items)  (also measured wellbeing using Warwick-Edinburgh Mental Wellbeing Scale, life satisfaction using Satisfaction with Life Scale, and contentment using Contentment with Life Assessment Scale.) | Post treatment follow-up (unspecified point after the 2 years of the intervention) | Post-test DJGLS-6 scores for the intervention group were reported to be significantly higher than the control group (mean difference = 2.4 , *p* =.001, *d* =2.43) and were also higher than their own  pre-test scores (mean difference = 2.54, *p* =.001, *d* =2.47). This was expressed as an indication of lower loneliness although the DJGLS uses high scores to denote greater loneliness. The author was unable to explain the apparent discrepancies.  The trial reported a significant difference in loneliness scores between intervention group and control group post-intervention (*t* = 21.77; *p* = .001, *d* = 2.43).  Table 3 suggests that loneliness scores increased significantly in the meditation group but not the control group, and that there was no difference in loneliness scores at pre-test but significantly higher loneliness scores in the intervention group at post-test (Table 3). The study reported group changes over time in a single model but did not specify the direction of change.  However, given the issues over interpretation of the poles of the DJGS we cannot infer evidence of effectiveness from this study. |
| O’Day et al., 2021 (USA) | RCT (3 groups) | Adults with social anxiety disorder (SAD) seeking treatment and scoring greater than 60 on the Liebowitz Social Anxiety Scale-Self Report (LSAS-SR) and met criteria for DSM-IV generalised Social Anxiety Disorder based on the Anxiety Disorder Interview Schedule for DSM-IV  Intervention group:  Mean age: 29.90 year (7.61)  Female: 55.6%  White: 38.9%  Asian: 36.1%  Latino: 19.4%  African American:2.8%  Multiracial: 2.8%  Single: 63.9%  Married: 22.2%  Living with partner: 13.9%  Control group:  Mean age: 34.06 year (7.78)  Female: 55.6%  White: 41.7%  Asian: 38.9%  Latino: 2.8%  American Indian: 2.8%  Multiracial: 13.9%  Single: 50.0%  Married: 44.4%  Living with partner: 2.8%  Not reported: 2.8% | 108 | Mindfulness based stress reduction therapy face-to-face group therapy  Twelve weekly 2.5 hour sessions with workbook to guide mindfulness practice | Waiting list | *UCLA-8 Loneliness Scale (ULS-8)  (also measured social anxiety using *Liebowitz Social Anxiety Scale-Self-Report* (LSAS-SR)) | End of treatment follow-up (3 months)  Post treatment follow-up (6, 9, 12 months) | Loneliness scores decreased for MBSR versus WL (*t* (103.82) = 3.57, *p* = .001, *d* = 0.34). There was no significant difference in loneliness between CBT and mindfulness-based stress reduction at post-treatment (*t* (105.86) = 0.53, *p* = .60, *d* = 0.05). There were no differences between treatments at post-treatment or during follow-up period (Cohen’s *d* = 0.05, *p* = .60). |
| **Approaches based on Social Identity Theory (*n* total studies =1)** | | | | | | | | |
| Haslam et al., 2019  (Australia) | RCT | Lonely adults diagnosed with a mental illness  Mean age: 31 years  Female: 64% | 120 | Groups4Health – group intervention focused on building and maintaining positive social group identifications  Delivered for 2 months | Treatment as usual (including evidence-based pharmacological or psychological treatment for depression or other mental health problems) | Roberts UCLA Loneliness Scale (RULS-8)  (8 items) | Post treatment Follow-up  (2 months)  4 months from baseline | The intervention group reported a significant decrease in loneliness  *(t* (97.0) =7.27, *p=*.001), but this was not observed in the TAU group (*t* (90.8) =2.23, *p =*.098).  There was a significantly greater decline in loneliness scores in the G4H group; an average of 3.83 points (*SE =* 0.53, *d = -*1.04) versus 1.23 points in the control group (*SE =* 0.55, *d* = -0.33). |
| **Approaches based on Self-Management of Well-being (SMW) theory (*n* total studies =1)** | | | | | | | | |
| Kremers et al., 2006 (Netherlands) | RCT | Lonely single women aged 55+  Intervention group:  Mean age 62.8 years (SD=6.4)  Control group: 65.2 (SD=7.6)  Female: 100% | 142 | Group self-management and well-being intervention  Six weeks | No information given | *De Jong Gierveld Loneliness scale (11 items)  (also measured self-management using the Self-Management Ability  Scale,  wellbeing using the Social Production Function Index  Level Scale) | End of treatment follow-up  (6 weeks)  Post treatment follow-up  (6 months) | Reduced loneliness in both groups, although statistical tests were presented for reductions within each group rather than group comparisons.  At the end of treatment, there was a significant reduction in the intervention group (*t* =89.0, *p* <0.01, r= -.31) and in the control group (*t* = 404.5, *p* <0.05, r = -.20), but the text reported no significant difference in intervention group compared to control group.  On loneliness sub-scales, the intervention group appeared to have significantly lower scores for social loneliness post-intervention but with no significant reduction in the control group, but again no formal group comparison statistical test was presented.  At six months follow-up there were no apparent group differences on loneliness, but again no formal group comparison statistical test was presented. |
| **Behavioural Activation (*n* total studies =1)** | | | | | | | | |
| Choi et al, 2020 (USA) | RCT | Housebound older adults (50+) experiencing loneliness  Mean age: 74 years  Female: 62% | 89 | Video conferenced, lay-coach facilitated,  short-term behavioral activation (Tele-BA) delivered for 1 hour a week for 5 weeks via a video-conference session | Video conferenced Friendly visit (Tele-FV) delivered for 1 hour a week for 5 weeks via a video-conference session | *8-item PROMIS  (Patient-Reported Outcomes Measurement Information System) Social Isolation Scale (PROMIS-L)  (also measured: Objective Social Isolation using the 4-item Social  Interaction Subscale of the Duke Social Support Index  (DSSI-I); Subjective Satisfaction with Social Support using the  6-item Social Satisfaction Subscale of the Duke Social  Support Index (DSSI-S)) | Six weeks and 12 weeks after intervention | Tele-BA participants reported  higher levels of  satisfaction with  social support  (*t* (82) = 2.00, *p* = .049) and lower levels of loneliness  (*t* (81) = -3.08, *p* = .003).  Effect sizes showed that Tele-BA had a medium effect on reducing loneliness and small-to-medium effects on the other outcome measures, all in the expected directions. |
| **Reminiscence therapy (*n* total studies =3)** | | | | | | | | |
| Chiang et al., 2010  (Taiwan) | RCT | Institutionalized males aged 65 years and over  Mean age: 77.2 | 130 | Reminiscence therapy  8 sessions over 8 weeks | Waiting list | *Revised UCLA loneliness scale (RULS-V3)  (20 items)  (also measured: depression using Center for epidemiological studies depression scale  (CES-D), psychological wellbeing using the Symptoms Checklist-90-R (SCL-90-R), and cognition using the  Mini-Mental State Examination (MMSE) | End of treatment follow-up (8 weeks)  Post treatment Follow-up  (3 months) | Loneliness was significantly reduced in intervention group compared to control group at follow-up  (*z* = -22.75, *p* <.0001). |
| Li et al., 2022 (China) | RCT | Individuals aged 60 years or older, who had previously lived with someone but had lived alone for at least one year.  Intervention group:  Mean age: 65.20 (SD=2.60)  Female: 60%  Went to high school or above: 13.3%  Married (divorced): 10% Married (widowed): 83% Unmarried: 6.7%  Control group:  Mean age:65.56 (SD=2.66)  Female: 66.7%  Went to high school or above: 13.3%  Married (divorced): 6.8%  Married (widowed): 90%  Unmarried: 3.3% | 60 | Group reminiscence therapy based on Chinese traditional festival activities (CTFA-GRT). Eight half-day sessions of group reminiscence therapy and activities based on Chinese traditional festivals | Waitlist control | *UCLA loneliness scale (UCLA-LS)  (also measured perceived stress using the Perceived Stress Scale (PSS)) | End of treatment follow-up (8 months)  Post-treatment follow-up ( 3 months after end of treatment) | The intervention group showed significantly lower scores on loneliness at T_1_ and T2 (three months and eight months after end of intervention).  At the post-test level after eight months (T2), a statistically significant primary effect for the group factor was observed for loneliness (*F* = 24.133, *p* < .001). A statistically significant primary effect for the time factor was also observed for loneliness (*F* = 46.202, *p* < 0.001).  After three months (T2), the group factor had a significant effect on loneliness (*F* = 36.584 -65.756, *p* < .001), meaning the intervention persisted for three months.  There were statistically significant differences (*F* = 58.521-143.214, *p* < .001) in the intervention group before and after the intervention for loneliness. |
| Ren et al., 2021 (China) | RCT | Individuals who were 60 years or older, with a GDS screening score between 10 and 25, with normal perception and language communication skills, and who were able to participate in physical exercise.  Intervention group:  Aged 60-69: 32  Aged 70-79: 16  Aged 80 or above: 12  Female: 28  With spouse: 45  Without spouse: 15  Control group:  Aged 60-69: 30  Aged 70-79: 20  Aged 80 or above: 11  Female: 31  With spouse: 42  Without spouse: 19 | 130 (121 analysed) | Routine community health education (4 lectures) + group reminiscence therapy (once a week for 50-60 minutes) + physical exercise (Taijiquan) | Routine community health education for 8 weeks (4 lectures) | *ULS Loneliness Scale  (also measured self-efficacy and life scheme using Spirituality Index of Well-Being (SIWB), and adaptability and resilience using Brief Resilience Scale (BRS)) | Post-treatment follow-up (1 month) | At 1 month follow-up, the loneliness scores decreased in both groups, and the difference with their respective value before the intervention was statistically significant (*p* < .001). The effect was better in the experimental group than in the control group, and the difference was statistically significant (*t* = 2.008 and 0.047, *p* = .047 and .001, respectively). |
| **Interpersonal psychotherapy (*n* intervention trials= 1)** | | | | | | | | |
| Käll et al., 2021 (Sweden) | RCT (3 groups) | Adults experiencing distress due to loneliness  Mean age: 47.5 years  Female: 75.9%  69.4% had a university degree  76% reported their civil status as either single (52.4%), divorced (17.1%), or widowed (6.5%). | 170 | Two Interventions: internet-based CBT  Internet-based IPT | Waiting list control | UCLA-Loneliness Scale-Version 3  (also measured depressive symptoms using the Patient Health Questionnaire-9 (PHQ-9), symptoms of social anxiety using Social Interaction Anxiety (SIAS), symptoms of generalised anxiety using Generalised Anxiety Disorder 7-item scale, quality of life measured using Bruusviken Brief Quality of Life Scale (BBQ)) | End of treatment (10 weeks)  Post-treatment follow-up (4 months) | IPT group did not exhibit a statistically significant decrease in loneliness during the study duration compared to the wait list control group (*b* = –1.36; 99% CI [ –4.19, 1.48]; *SE* = 1.44; *p* = 1; *d* = 0.18). The analysis of post-treatment to four-month follow-up demonstrated a non-significant decrease in loneliness (*p* = 0.15) |
| **Orem’s self-care deficit theory (*n* total studies =1)** | | | | | | | | |
| Ökten & Özer, 2022 (Turkey) | RCT | Patients (aged 18 or older) diagnosed with colorectal cancer receiving chemotherapy treatment  Intervention group:  Mean age: 60.38 years (SD=8.58)  Female: 20.8%  Single: 12.5%  Married: 87.5%  Employed: 37.5%  Control group:  Mean age: 57.52 years (SD = 12.65)  Female: 30.4%  Single: 8.7%  Married: 91.3%  Employed: 52.2% | 47 | Orem’s self-care deficit theory educational group-based intervention with telephone follow-ups  One educational session for 45-50 minutes followed by three 10–15-minute phone calls | Treatment as usual (brief education of the side effects of treatment was provided before chemotherapy, followed by the treatment itself) | *UCLA Loneliness Scale 3 translated to Turkish  (also measured self-care agency using the Exercise of self-care agency translated into Turkish, physical well-being, social well-being, and psychological well-being using Nightingale symptom assessment scale, state and trait anxiety using State-Trait Anxiety Inventory (STAI-S, STAI-T) translated into Turkish) | No information given | There was a statistically significant difference between the mean loneliness scores of the intervention and control groups (*F* = 74.50, *p* < .001), with the intervention group’s mean loneliness scores being significantly lower than those of the control group at both assessments (Time 1 and time 2: *t* = −5.081, *p* < .001; *t* = −7.098, *p* < .001, respectively). |
| **Expressive writing and imagined interaction theory (*n* total studies =1)** | | | | | | | | |
| Zhang et al., 2023 (USA) | RCT (3 groups) | Adults scoring 79 or lower on the Mental Health Inventory  Intervention group (rehearsal):  Mean age: 24.4 years (SD = 9.3, range = 18-63)  Female: 64.4%  White: 51.1%  African American: 4.4%  Asian: 26.7%  Latino: 13.3%  Others: 4.4%  Intervention group (replay):  Mean age: 26.0 years (SD = 12.1, range = 18-74)  Female: 61.4%  White: 40.9%  African American: 2.3%  Asian: 29.5%  Latino: 27.3%  Control group:  Mean age: 22.8 years (SD = 8.5, range =18-72)  Female: 62.8%  White: 34.9%  African American: 7%  Asian: 37.2%  Latino: 16.3%  Others: 4.7% | 132 | Individual expressive writing digital intervention that involved replaying  previous interactions or rehearsal for future interactions  Four days of writing for 15 minutes | Control group (writing about a neutral topic) | *3 item version of the Revised UCLA Loneliness scale  (also measured psychological distress using the Mental Health Inventory (MHI), perceived stress in life situations using the Perceived Stress scale (PSS), social and work-related adjustment to stressors using Work and Social Adjustment scale (WSAS), coping behaviours using Brief COPE, symptoms of anxiety and depression using Patient Reported Outcomes Measurement Information System) | End of treatment follow up (4 days)  Post-treatment follow-up (1 month) | Rehearsal group show a consistent decrease in loneliness post writing (rehearsal group Mean (*SD*): T0= 7.2 (1.6), T2= 7.1 (1.7); replay group: T0= 6.6 (1.6) , T2= 6.8 (1.5))  and at 1-month follow-up (rehearsal Mean (*SD*)= 7.0 (1.6) ; replay group= 6.4 (1.7)) than the other two groups when visually assessing the mean estimates. No significant effects of time (*F* = 1.748, **η^2^** = 0.013) or group by time effects ( *F* =0.485, **η^2^** =0.008) were found for loneliness. |
| **Logotherapy (*n* total studies =1)** | | | | | | | | |
| Heidary Heshmati, & Hayes, 2023 (Iran) | RCT | Patients with advanced-stage cancer  Intervention group:  Mean age: 53.93  Female: 48.38%  Control Group:  Mean age: 52.56  Female: 53.13% | 63 | Logotherapy group therapy delivered in 10 2-hour sessions over 10 weeks, participants divided into 4 groups of 8 patients. Aimed to explore the effect of group logotherapy on death anxiety and existential loneliness. | Waitlist control, patients were asked not to participate in nonobligatory spiritual or religious ceremonies. | *19-item questionnaire measuring existential loneliness (Existential Loneliness Questionnaire [ELQ])  (also measured anxiety about death using the Templer’s Death Anxiety Scale (DAS)) | Post-treatment follow up (immediately after the last intervention session in week 10) | Post-test existential loneliness scores were significantly lower in the logotherapy group than the waitlist controls (*t* (61) = -5.19, *p* < .001).  Simple effects tests showed significantly lower existential loneliness at posttest compared to pretest in logotherapy group (*t* (30) = -8.79, *p* < .001). In the waitlist control, scores were significantly higher at post-test compared to pretest (*t* (31) = 3.65, *p* < .001) |
|  | | | | | | | | |

* One of several primary outcomes listed
