# Supplementary material for: Investigating the effectiveness of interventions intended to reduce loneliness using psychological strategies and a theory of change: a systematic review of interventional studies and meta-analysis
Source: BMC Psychol. 2025 Dec 12;14:131. doi: 10.1186/s40359-025-03639-3 (PMC12857015; doi:10.1186/s40359-025-03639-3)
Supplement: Supplementary file 6 — Additional file 6: Supplementary Table 2. Details of interventions and their theory of change classified by intervention type. [file 40359_2025_3639_MOESM6_ESM.docx]

**Supplementary Table 2**

*Details of interventions and their* *theory of change* *classified by intervention type (n=22)*

| **Study** | **Intervention** | **Psychological factors targeted** | **Intervention processes and actions** | **Theory of Change** | **Intervention Format**  **& Intensity (and mode of delivery)** |
| --- | --- | --- | --- | --- | --- |
| **Cognitive-behavioural approaches (*n* total studies =10)** | | | | | |
| Conoley & Garber, 1985 (USA) | Reframing intervention to increase attributions of the controllability of loneliness | Attributional styles: perceptions of  loneliness | Reframing loneliness | The approach aimed to encourage participants to reframe their experience of loneliness to be under their own control and potentially a positive aspect of their own personal development, rather than a result of interpersonal failures that they may attribute to themselves. The approach intended to reduce loneliness by breaking a reinforcing cycle of maladaptive approaches to dealing with loneliness and loneliness itself | Two sessions, lasting 30 minutes, held a week apart  **Face-to-face individual intervention** |
| McWhirter & Horan, 1996 (USA) | Group counselling meetings utilising cognitive restructuring and behavioural techniques to address a) intimate relationships, b) social relationships, c) both of these | Attributional styles | intimate loneliness intervention*:* cognitive and behavioural techniques focused on establishing and maintaining intimate relationships  social loneliness intervention: cognitive restructuring,  role-plays, home-work in different loneliness  domains | Intimate loneliness was addressed through emphasising the importance of establishing and maintaining close relationships in reducing loneliness. Role play and homework tasks were used to increase practical social skills and confidence in order to help form close relationships  Social loneliness was addressed through modelling, role play and homework assignments to help participants restructure maladaptive cognitions around loneliness and social relationships. The approach also aimed to reduce stress in small social situations so that participants feel more confident with others and therefore more likely to form friendships | Six sessions, lasting 2 hours each, held a week apart over six weeks.  **Face-to-face group intervention.** |
| Theeke et al., 2016 (USA) | Group sessions focusing on perceived belonging, perception of relationships, coping with loneliness and identifying meaning in peoples experience and self-assessment of maladaptive cognitions, behaviours and emotions | Rethinking the  experience of  loneliness to  enhance meaning  and facilitate moving  forward | Targeting stress  mechanism linking health and loneliness | Session 1 of the programme encouraged participants to think about their self over their life, and their feelings of belonging and loneliness, to help facilitate identification of automatic thoughts they experience when thinking about loneliness. Session 2 focused on participants re-connecting with their self in relation to others and their community. Sessions 3, 4 and 5 focused on the conceptualisation of loneliness, how participants cope with it and re-capping.  The approach is likely to work through helping participants to rethink and reframe their perception of loneliness and maladaptive cognitions, and to enhance meaning. Acquiring skills and the group format may improve self-esteem and social confidence leading to reduced stress | Five sessions, lasting 2 hours each, held a week apart over five weeks.  **Face-to-face group intervention** |
| Cohen-Mansfield et al., 2018 (Israel) | I-SOCIAL intervention focused on identifying and addressing personal barriers to social integration.  The intervention included:  (1) identifying the barriers for the specific person; (2) up to ten individual meetings with an activities counselor, which focused on helping the person address personal barriers to social integration and included discussions concerning options for social contacts as well as using techniques and local resources to tackle the barriers (e.g., undertaking a mapping of social opportunities in the neighborhood using resources from local governments and senior centers); and (3) up to  seven group sessions of participants and the activities counselors were  held in order to provide opportunities to increase social competence by practising social skills within a protected setting, and as a venue to  discuss barriers and ways to address them | Addressing personal barriers to social integration, such as low social self-efficacy | Activity facilitators used cognitive-behavioural principles in sessions to remove barriers to social engagement | The I-SOCIAL intervention focused on addressing psychosocial factors that produce and maintain loneliness. Group sessions included opportunities to practice social skills with others, increasing self-efficacy, social competence, and confidence that might have previously prevented participants from forming meaningful relationships. Individual sessions with a counsellor focused on identifying individual psychosocial barriers to positive social relationships, and practical steps to overcome them. Overall, the approach may increase awareness of potential social opportunities, and the self-efficacy and confidence to attend to them ultimately reducing loneliness through engaging in more social behaviours | Up to 10 face-to-face individual sessions, and up to 7 group sessions, over a 6-month period.  **Face-to-face intervention held individually and in groups** |
| Jarvis Jarvis, Padmanabhanunni, & Chipps, 2019 (South Africa) | Low-intensity cognitive-behavioural therapy. Face-to-face psycho-education and individualised positively worded messages addressing maladaptive cognitions delivered via a mobile messaging app (WhatsApp) | Psycho-education  Maladaptive cognitions linked to loneliness, and reflecting on the cognitive distortion | Address  maladaptive  cognitions | Positively framed text messages were sent to participants to give them the opportunity to reflect on how their maladaptive cognitions around loneliness may be influencing their behaviours and emotions. By altering maladaptive social cognitions, participants may feel less alienated, as socially different or an outsider, and have an increased sense of belonging | Three-month intervention with a 2 week 4-session blended face-to-face and digital program.  **Individual intervention delivered digitally and face-to-face** |
| Käll et al., 2019 (Sweden) | Internet-based Cognitive Behaviour Therapy with modules on psycho-education and assignments linked around the experience of loneliness | Cognitions and  behaviours  associated with  loneliness | Formulation, cognitive  restructuring, behavioural experiments,  behavioural activation | Three modules focused on cognitive restructuring, which aimed to identify and deal with maladaptive cognitions linked to the participants loneliness. A further three modules utilised behavioural activation to increase participation in social events and increase the perceived quality of existing relationships. Interventions in these modules aimed to increase help-seeking behaviours and reduce social withdrawal that can lead to loneliness. Overall, the therapy is likely to reduce loneliness through reducing negative cognitions and rumination, increasing social confidence and self-esteem leading to a higher number of social contacts and opportunities to make and maintain meaningful relationships | Eight-week program consisting of 8 modules, with one module completed each week.  **Individual digital intervention** |
| Breuhlman-Senecal et al, 2020 (USA) | Nod: a mobile app intended to incorporate positive psychology, mindfulness-based self-compassion, and cognitive-behavioural skill-building exercises to address loneliness among first-year  college students | Negative self and social cognitions | Encourage a growth mindset towards social connection building  Reduce self-criticism | “Social challenge” content focused on building participants social skills, which would help enable them to effectively build social connections with others.  “Reflections” content aimed to amplify and prolong positive emotions following social events, or to reframe negative thoughts about social events via meditation or reappraisal. The app could therefore reduce loneliness through addressing cognitive biases linked to social interactions such as fears of judgement and rejection, therefore improving social connections due to improved confidence. The app aimed to reinforce the positive experience of social interactions therefore making the user more likely to engage in social behaviours in the future, therefore increasing social behaviours and reducing the likelihood of feeling lonely | Individual intervention delivered digitally via a Smartphone app over a 4-week period.  **Individual digital intervention.** |
| Shapira et al, 2021 (Israel) | Cognitive-behavioural based digital group intervention consisting of online guided group sessions delivered via an online video platform (Zoom) and moderated by clinical social workers and intended to help participants acquire cognitive and behavioural skills related to coping with the COVID-19 pandemic and to mitigate the potential mental health effects of social isolation (e.g. loneliness and associated depression_ | Identifying non-adaptive cognitive schemas and cognitive restructuring to promote better coping  Constructing positive  self-talk  Mindfulness techniques for  distancing thoughts and sensations | More adaptive coping abilities, and improved resilience to negative beliefs and appraisals | Group discussions provided social contact during a period of isolation owing to the Covid-19 pandemic. The facilitators aimed to help participants identify maladaptive cognitive schemas, and to restructure them in a positive way to reduce negative affect and feelings of isolation. Mindfulness techniques were taught that could distract from negative feelings of loneliness | Online group intervention delivered twice weekly over 3 and a half weeks (7 sessions in total) using an online video platform (Zoom).  **Group digital intervention.** |
| Käll et al 2021 (Sweden) | Cognitive-behavioural internet-based intervention about concepts of valued social interactions. Participants completed assignments and were contacted by CBT therapists via the messaging function of a platform. The intervention was based on concepts of valued social intervention that would counteract feelings of loneliness. This involved psychoeducation, behavioural activation to foster change, strategies to deal with barriers (e.g. maladaptive cognitive schemas and social skills training). | Identifying maladaptive cognitive schemas and cognitive restructuring to promote social contact. | The modules involved identifying treatment goals, developing adaptive coping strategies, and psychoeducation and application of behavioural activation, social skills training. The modules contained interactive assignments, text and images related to loneliness and how to deal with it. | The modules centred on identifying valued social contact that aligned with participant’s beliefs and treatment goals and provided psychoeducation and behavioural activation. Behavioural activation strategies were used to foster change towards valued social contact and reduce loneliness. Maladaptive cognitions linked to loneliness and social anxiety were addressed using cognitive restructuring intervention and behavioural experiments to reframe negative cognitions into positive cognitions. Overall, addressing negative cognitions associated with loneliness is likely to reduce loneliness through increasing valued social contact and improving quality of existing relationships, cognitive restructuring negative thoughts and reducing social anxiety. | Internet based intervention delivered over 9 weeks, completing one assignment per week. Participants received weekly feedback and contact the therapist via an online messaging platform.    **Individual digital intervention** |
| O’Day et al., 2021 (USA) | A cognitive behavioural group therapy delivered by PhD-level psychologist. Treatment components consisted of psychoeducation, cognitive restructuring, in-vivo exposure, and relapse prevention | Identify negative automatic thoughts and engage in feared social situations and challenge negative thoughts | Learning cognitive restructuring skills to challenge negative thoughts and in-vivo exposure to feared social situation and relapse prevention | This approach aimed to target negative thoughts and social anxiety symptoms by learning cognitive restructuring skills to challenge and dispute negative thoughts and engage in in-vivo exposure to feared negative situations that would elicit negative thoughts. This would reduce maladaptive social cognitions and promote approach rather than avoidance of social situations which would reduce feelings of loneliness | Twelve weekly 2 hour and a half face-to-face group session.  **Face-to-face group intervention, with individual homework assignments** |
| **Mindfulness (*n* total studies =4)** | | | | | |
| Creswell et al., 2012 (USA) | Mindfulness Based Stress Reduction. Sessions focussed on meditation exercises, mindful yoga and group discussions. | Distance from  cognitions relating to  social threat/distress  and negative affect | Meditation to change relationship with loneliness | Mindfulness could distract from participants’ subjective perception of isolation and reduce distress and negative affect which in turn may decrease loneliness | Eight weekly 2 hour face-to-face group sessions, a one day retreat in the 6^th^ or 7^th^ week, and 30 minutes of daily individual mindfulness meditation at home, over a period of 8-weeks.  **Face-to-face group intervention, with individual homework** |
| Zhang et al., 2016 (China) | Mindfulness-based Cognitive Therapy with psycho-education on loneliness and mindfulness exercises | Maladaptive cognitive patterns/ de-identify with perceived social threat | Meditation to change relationship with loneliness; psychoeducation about loneliness | Increased awareness of maladaptive thinking patterns could lead to improved emotion regulation, self-acceptance, self-care and a positive outlook on the future, which may enhance the capacities of participants to disengage from perceived social threats thus reducing feelings of loneliness | Eight two-hour face-to-face group sessions delivered weekly  **Face-to-face group intervention.** |
| Pandya, 2021  (India, Nepal, Burma, Sri Lanka) | Group meditation program including mindfulness training focused on centering, concentration and stillness | Social cognitions such as automatic negative thoughts | Training in perspective taking and empathy and ability to identify automatic negative thoughts about  others and about social interactions to target and address faulty behavioural confirmation processes | Meditation programme focused on centering, concentration and stillness to enhance participants emotional regulation, self-efficacy and self-compassion, in turn influencing social cognition and negative thoughts and feelings associated with an internal perception of one’s own loneliness | 45-minute guided group meditation lessons conducted once a week for 2 years at a designated city location (up to 100 sessions on two sites; group size range =21–28; mean group size= 24.5, SD=4.95) by instructors, supplemented by individual meditation practice at home once a week prior to the next class and provision of self-help literature  **Face-to-face group intervention, with individual homework** |
| O’Day et al., 2021 (USA) | Mindfulness-based Stress Reduction group therapy intervention administered by master level certified instructor. Meditation and focusing on acceptance of negative emotions and thoughts | Negative and maladaptive anxious thoughts, feelings and behaviours | Mindfulness training in noticing and non-judgementally accepting maladaptive social cognitions | The intervention focused on the use of mindfulness and acceptance strategies to notice and non-judgementally accept anxious feelings, thoughts and behaviours. This would help clients examine their maladaptive anxious thoughts and social cognitions which then reduces loneliness | Twelve weekly 2 hour and a half face-to-face group session.  **Face-to-face group intervention, with a workbook to guide mindfulness practice** |
| **Social identity theory** | | | | | |
| Haslam et al., 2019 (Australia) | Groups 4 Health (G4H) is a manualised 5-module psychological intervention that targets the development and maintenance of social group relationships to treat psychological distress arising from social isolation. The program consists of 5 modules (Schooling, Scoping, Sourcing, Scaffolding, Sustaining) which progress through psycho-education on social groups, identifying current social networks, brainstorming ways of extending group memberships, and reinforcing behaviour change by celebrating successes | Social identity | Social group  belonging | The intervention targets the development and maintenance of positive social group relationships. Psychoeducation and mapping exercises increase participants’ understanding of their own social groups and how to enhance them. Further exercises including identifying ways to reconnect with existing social groups and how to overcome any potential barriers. Overall, the intervention aims to strengthen an individual’s sense of social identity, improve their sense of psychological belonging, well-being and self-esteem, and reduce any psychological distress and social anxiety, ultimately reducing subjective feelings of loneliness and isolation | Five face-to-face group sessions lasting 90 minutes over a 2-month period.  **Face-to-face group intervention** |
| **Self-Management of Wellbeing theory** | | | | | |
| Kremers et al., 2006 (Netherlands) | Group intervention focused on improving six self-management abilities (taking initiatives, self-efficacy, investment behaviour, positive frame of mind, ensuring multifunctionality in resources, ensuring variety in resources) to achieve and maintain the resources that are needed to satisfy five basic dimensions of wellbeing (comfort, stimulation, affection, behavioural confirmation, and status). A specific focus on achieving and maintaining  friends satisfies the dimension of affection, as a means of improving well-being and reducing social and emotional loneliness | Self-management of abilities to develop personal resources, such as to make friends, maintain friendships, ensure multi-functional friendships (those fulfilling a range of needs), and to ensure variety (in having more than one friend) | Goal-setting based on the six key self-management abilities and self-assessment of steps made to meet those goals, including challenging negative thoughts and reframing them as positive thoughts, with paper records of progress towards goals | The intervention focuses on self-management abilities that are key to well-being. Participants are encouraged to reflect on basic needs they may be missing and how their needs could be met and achieved through activities or being with others, therefore fostering a mindset for social interaction, integration, and embeddedness. Through the improvement of self-management abilities participants are able to more effectively maintain friendships. Throughout participants were told to replace any negative thoughts with positive ones. Together, these potentially improve affect and well-being and therefore reducing feelings of social and emotional loneliness | Six face-to-face group meetings, in a group size of 8-12 people, lasting 2.5 hours each, over 6 consecutive weeks, with two female facilitators  **Face-to-face group intervention** |
| **Behavioural Activation** | | | | | |
| Choi et al, 2020 (USA) | Psychoeducation content focused on social connectedness, with lay coaches working with participants to identify and schedule rewarding social engagement and use appropriate strategies to reduce barriers to social connectedness | Positive reinforcement of wellness-promoting behaviours (e.g. engaging in meaningful life activities aligned with personal values) and reduction of depressive behaviours | Psychoeducation (education and coaching) content to identify and schedule values-based, rewarding social engagement and activities and to use strategies to reduce and problem-solve barriers to social connectedness. | Participants learn about the importance of social connectedness through psychoeducational content and positive reinforcement of social engagement, as well as problem-solving any factors reinforcing their avoidance of social engagement. This improves awareness of any tendency to avoid the aversive thoughts, feelings or external situations associated with social engagement, and how this might reinforce loneliness. Redressing avoidance serves to activate social engagement and reduce rumination and lost opportunities for pleasurable social engagements. | Five sessions of Tele-BA delivered weekly by lay coaches via videoconference over a five week period.  **Individual digital intervention** |
| **Reminiscence therapy (*n* total studies =3)** | | | | | |
| Chiang et al., 2010 (Taiwan) | Reminiscence group therapy. Focusing on awareness of feelings, past and current relationships, positive strengths and goals | Sense of self/ well-being/ increased awareness of feelings | Ego-integrity, mastery,  meaning of life, and social integration | The approach was intended to improve cognitive functioning in relation to comprehension skills and boost self-esteem and sense of self to address feelings of loneliness, depression, and hopelessness. The group approach provided a rewarding sense of belonging, with the sharing of memories stimulating friendship, cohesion, and acceptance between participants. The pleasurable activity of recalling past events, either positive, sad or profound, is also seen to promote wellbeing, health, and positive affect.  Authors state in discussion re the group format:  “The study gave the participants a chance to interact with people rather than remaining alone for the whole time. The sharing and positive feedback among  participants’ stimulated friendships and a greater  personal understanding for each other, giving them  a sense of belonging to a group and acceptance by the  group. By learning about others’ lives, the participants  realized that every life was unique and interesting, even  if there were some sad or frustrated stories. The group  therapy built a strong sense of belonging and cohesion  among participants that helped to ease feelings of  loneliness. The results further indicate that reminiscence  can help ease the pain of isolation and loneliness.  Memory is used as a therapeutic intervention to help validate a sense of self.” | Eight weekly sessions, lasting 90 minutes each over an 8 week period.  **Face-to-face group intervention** |
| Li et al., 2022 (China) | Group reminiscence therapy based on Chinese traditional festival activities (CTFA-GRT). Sessions aimed at establishing a relationship of trust, getting to know each other, enhancing positive experience, venting feelings and showing themselves, recalling maternal love, sharing health knowledge, and actively facing life | Encourage participants to express themselves more actively and share their feelings, while targeting their sense of belonging | Through group reminiscence therapy and activities, sense of belonging and social connectedness is increased | CTFA-GRT encourages individuals to express themselves and share their feelings, thereby reducing loneliness. Additionally, the group format of it and the common theme (Chinese traditional festival activities) increases sense of belonging and social connected. This in turn reduces loneliness | Monthly session for eight months. It then involves reminiscence and activities based on the session’s festivity. Homework is assigned  **Face-to-face group intervention. Participants use WeChat to complete assignments and leave messages** |
| Ren et al., 2021 (China) | The intervention included routine community health education and group reminiscence therapy, as well as Taijiquan exercises for 8 weeks | Improve sense of support, belonging, and social connection, as well as relieve the negative emotions | Group reminiscence therapy among people with similar backgrounds to increase their sense of social support, connection and belongingness | The reminiscence of the elderly with similar backgrounds relieves the elderly’s original negative emotions. Additionally, the group atmosphere enhances the sense of social connection among the elderly, leads them to feel understood and respected, experience more social support and perceive sense of belonging, thus reducing their loneliness | Weekly group reminiscence therapy (50-60 minutes each time) for 8 weeks. In addition, a total of 4 routine health lectures (one every two weeks) and Taijiquan exercise (3 times a week, 45 minutes each time).  **Face-to-face group intervention.** |
| **Interpersonal psychotherapy** | | | | | |
| Käll et al 2021 (Sweden) | Internet based IPT focusing on the link between mood and interpersonal interactions | Targeting maladaptive cognitive patterns and behaviours and interpersonal factors and their impact on interpersonal events. Emotional processing of feelings associated with loneliness | Address interpersonal factors (e.g. maladaptive behavioural and cognitive patterns) and interpersonal conflict (e.g. adverse interpersonal events and lack of social support).  Focus on one of four areas: conflict, grief, interpersonal deficits and role transition, and use communication analysis and experiential processing of feelings. The modules contained interactive assignments, text and images related to loneliness and how to deal with it | The approach proposed that loneliness and psychopathology results from adverse changes in one’ social network and subsequent lack of support. This intervention aimed to identify the impact of loneliness and reduce loneliness through addressing maladaptive cognitive patterns that result from interpersonal events and factors. The intervention focused on significant interpersonal events that may be the onset of loneliness (e.g. major disruption in social network or divorce/bereavement). Communication analysis and experiential exercise to promote emotional process of feeling related to loneliness were incorporated into the intervention | Internet based intervention delivered over 9 weeks, completing one assignment per week. Participants received weekly feedback and contact the therapist via an online messaging platform  **Individual digital intervention** |
| **Orem’s self-care deficit theory** | | | | | |
| Ökten & Özer, 2022 (Turkey) | An educational programme based on Orem’s self-care deficit theory to improve quality of life during chemotherapy. This programme includes an educational component on self-care behaviours for ways to manage symptoms | Self-care agency (patient’s knowledge and skills on symptom management) and self-care deficits (Inability to manage symptoms) | Improving self-care agency and skills through an educational component and follow up telephone calls to address experiences and problems identified to inform patient’s self-care plan | This approach aimed to teach Orem’s self-care deficit theory and focus on self-care and activities that are performed by individuals to maintain well-being and physical health. This would increase self-care activities which would then improve self-care agency. Self-care interventions aim to achieve a balance between loneliness and social interaction and suggests that improving coping with physical symptoms would contribute to improved quality of social relationships and reduces loneliness | One education session that lasted 45-50 minutes followed by three 10–15-minute phone calls. Participants were provided educational booklets  **Individual telephone based and education group-based intervention** |
| **Expressive writing and imagined interaction theory** | | | | | |
| Zhang et al., 2023 (USA) | A therapeutic intervention based on expressive writing and rehearsals either about previous interactions (replay conditions) or expressive writing and rehearsal about future imagined events (rehearsal condition). Both interventions are based on Pennebaker’s expressive writing paradigm and imagined interaction theory in which individuals mentally prepare and rehearse interactions and emotional experiences | Attentional processing and cognitive processing of past events that were traumatic and relate to mental health condition | Building cognitive scripts and narratives about previous past or ongoing mental health related stressor and desensitisation of trauma to improve ability to confront trauma or stressor | Expressive writing and replay of and reflection on previous traumatic experiences promotes awareness of previously avoided and inhibited memories and emotional experiences. This theory suggests that through writing about emotionally meaningful topics and replaying these topics, people are prepared to confront their trauma which would improve psychological health outcomes including loneliness. The mechanism of effect is through attentional processing and cognitive processing | Four days of writing for 15 minutes. Participants are sent a reminder to complete daily writing  **Individual digital intervention** |
| **Logotherapy** | | | | | |
| Heidary Heshmati, & Hayes, 2023 (Iran) | Group logotherapy is a meaning-oriented intervention to help individuals in the advanced stages of cancer reduce feelings of existential loneliness | Developing psychological resilience, sense of meaning in life and connectedness with the world | Identifying and exploring creative, experiential and attitude values in participants’ lives  Goal setting, fitting goals with values and planning for goal achievement | Participants learn about and are encouraged to make meaning of their lives and promote a sense of purpose through identifying values and setting goals.  Participants learn to address feelings of existential loneliness through sharing with the group, developing awareness of a common hardship and that they are not alone in their struggles. This allows each participant to feel more connected to the world around them and less alone. Sharing experiences may promote a sense of genuine interpersonal connectedness and thus reduce existential loneliness.  The group-based element may encourage sharing in problems caused by illness and group empathy, as well as forming relationships with others in the group which encourages exploring the meaning of life | 10 face-to-face sessions for 10 weeks, conducted by a qualified psychologist (with 15 years experience in this treatment). Each session lasted 2 hours, with around 8 participants in each group  **Group face-to-face intervention** |
