# Supplementary material for: Investigating the effectiveness of interventions intended to reduce loneliness using psychological strategies and a theory of change: a systematic review of interventional studies and meta-analysis
Source: BMC Psychol. 2025 Dec 12;14:131. doi: 10.1186/s40359-025-03639-3 (PMC12857015; doi:10.1186/s40359-025-03639-3)
Supplement: Supplementary file 7 — Additional file 7: Supplementary Table 3. Risk of bias in included studies classified by intervention type. [file 40359_2025_3639_MOESM7_ESM.docx]

**Supplementary Table 3**

*Risk of bias in included studies classified by intervention type (n=22)*

| **Study** | **Randomisation process** | **Deviations from intended interventions** | **Missing Outcome** | **Measurement of outcome** | **Selection of the reported results** | **Overall risk of bias** |
| --- | --- | --- | --- | --- | --- | --- |
| **Cognitive behavioural theory** | | | | | | |
| Conoley & Garber, 1985 | Some concerns | Some concerns | High | Low | Some concerns | High |
| McWhirter & Horan, 1996 | Some concerns | Some concerns | Low | High | Some concerns | High |
| Theeke et al., 2016 | Some concerns | Some concerns | High | Low | Some concerns | High |
| Cohen-Mansfield et al., 2018 | Some concerns | Low | Some concerns | Some concerns | High | High |
| Jarvis Padmanabhanunni, & Chipps, 2019 | Some Concerns | Some concerns | Low | Some concerns | Some concerns | Some concerns |
| Käll et al., 2020 | Low | Low | Low | Some concerns | Some concerns | Some concerns |
| Bruehlmann,-Senecal, 2020 | Low | Low | Low | Some concerns | Some concerns | Some concerns |
| Shapira et al, 2021 | Low | Low | Low | Some concerns | Some concerns | Some concerns |
| Käll et al., 2021 | Low | Some concerns | Low | Some concerns | Some concerns | Some concerns |
| **Mindfulness** | | | | | | |
| Cresswell et al., 2012 | Low | Low | Low | Low | Some concerns | Some concerns |
| Zhang et al., 2016 | High | Low | Some concerns | Some concerns | Some concerns | High |
| Pandya, 2021 | Low | Low | Low | High | Low | High |
| O’Day et al., 2021 | Low | Low | Low | High | Low | High |
| **Social identity theory** | | | | | | |
| Haslam et al, 2019 | Low | Low | Some concerns | Some concerns | Low | Some concerns |
| **Self-Management of Well-being theory** | | | | | | |
| Kremers et al., 2006 | Some concerns | Some concerns | High | Some concerns | Some concerns | High |
| **Behavioural Activation** | | | | | | |
| Choi et al 2020 | Low | Low | Low | Low | Some concerns | Some concerns |
| **Reminiscence therapy** | | | | | | |
| Chiang et al., 2010 | Low | Low | High | Some concerns | High | High |
| Li et al., 2022 | Low | Some concerns | Low | Low | Some concerns | Some concerns |
| Ren et al., 2021 | Some concerns | Some concerns | Low | Some concerns | Some concerns | High |
| **Orem’s self-care deficit theory** | | | | | | |
| Ökten & Özer., 2022 | Low | Some concerns | Low | Some concerns | Some concerns | Some concerns |
|  |  |  |  |  |  |  |
| **Expressive writing and imagined interaction theory** | | | | | | |
| Zhang et al., 2022 | Some concerns | Low | Low | Some concerns | Some concerns | Some concerns |
| **Logotherapy** | | | | | | |
| Heidary Heshmati, & Hayes, 2023 | Low | Some concerns | Low | Low | Some concerns | Some concerns |
